# Variable interplay of UV-induced DNA damage and repair at transcription factor binding sites

**DOI:** 10.1093/nar/gkaa1219

**Published:** 2020-12-21

**Authors:** Joan Frigola, Radhakrishnan Sabarinathan, Abel Gonzalez-Perez, Nuria Lopez-Bigas

**Affiliations:** Institute for Research in Biomedicine (IRB Barcelona), The Barcelona Institute of Science and Technology, Baldiri Reixac, 10, 08028 Barcelona, Spain; Thoracictumors and head and neck cancer group, Vall d’Hebron Institute of Oncology. Natzaret, 115-117, 08035, Barcelona, Spain; National Centre for Biological Sciences, Tata Institute of Fundamental Research, Bangalore 560065, India; Institute for Research in Biomedicine (IRB Barcelona), The Barcelona Institute of Science and Technology, Baldiri Reixac, 10, 08028 Barcelona, Spain; Research Program on Biomedical Informatics, Universitat Pompeu Fabra,Barcelona, Catalonia, Spain; Institute for Research in Biomedicine (IRB Barcelona), The Barcelona Institute of Science and Technology, Baldiri Reixac, 10, 08028 Barcelona, Spain; Research Program on Biomedical Informatics, Universitat Pompeu Fabra,Barcelona, Catalonia, Spain; Institució Catalana de Recerca i Estudis Avançats (ICREA), Barcelona, Spain

## Abstract

An abnormally high rate of UV-light related mutations appears at transcription factor binding sites (TFBS) across melanomas. The binding of transcription factors (TFs) to the DNA impairs the repair of UV-induced lesions and certain TFs have been shown to increase the rate of generation of these lesions at their binding sites. However, the precise contribution of these two elements to the increase in mutation rate at TFBS in these malignant cells is not understood. Here, exploiting nucleotide-resolution data, we computed the rate of formation and repair of UV-lesions within the binding sites of TFs of different families. We observed, at certain dipyrimidine positions within the binding site of TFs in the Tryptophan Cluster family, an increased rate of formation of UV-induced lesions, corroborating previous studies. Nevertheless, across most families of TFs, the observed increased mutation rate within the entire DNA region covered by the protein results from the decreased repair efficiency. While the rate of mutations across all TFBS does not agree with the amount of UV-induced lesions observed immediately after UV exposure, it strongly agrees with that observed after 48 h. This corroborates the determinant role of the impaired repair in the observed increase of mutation rate.

## INTRODUCTION

Living cells are exposed to agents with the potential to damage their DNA (1–3). The lesions that result from this damage which are not dealt with by repair mechanisms at the time of replication may result in mutations (4–6). A repertoire of such repair programs is involved in the correction of different types of lesions (7–9). For example, the exposure to UV light, a well known mutagen, may cause adjacent pyrimidines to form abnormal covalent bonds creating dipyrimidine adducts, the most abundant type of which are cyclobutane pyrimidine dimers (CPDs) (10). These lesions are preferentially detected and repaired by the nucleotide excision repair (NER) (3) machinery. Unrepaired bulky CPDs may block the advance of the replication fork, thus triggering the recruitment of translesion polymerases, capable of bypassing the blockage, sometimes at the cost of incorrect nucleotide insertion (11). In the case of UV-induced damage, adenines may be inserted opposite CPDs. Moreover, cytosines in CPDs are more prone to undergo spontaneous deamination than cytosines in B-DNA (12). Both processes thus result in C-G>T-A mutations in cytosines within dipyrimidine contexts, leaving a distinctive mutational pattern known as the UV mutational signature (COSMIC signatures 7a-c) (13,14).

The distribution of mutations along the genome that results from DNA-damage processes, such as exposure to UV light varies depending on both, large and small-scale genomic features (6,15). For example, chromatin regions with different replication time or genes with different levels of expression (16,17) receive different rates of mutations. Chromatin features that affect the mutation rate at the local scale include nucleosomes (18) and active transcription factor binding sites (TFBS) (19) among others. The rate of mutations in TFBS in certain cell types, such as melanocytes (and the derived tumor type melanomas), is abnormally high in comparison to that observed in their flanking regions (19). Several studies have reported an unexpected increase of the mutations in active TFBS in melanomas linked to a reduced accessibility of the NER machinery to these regions (19,20). More recently, an abnormal increase in the formation of CPDs in the active binding sites of the ETS TFs has been reported as a cause of the increased mutation rate in their binding sites (21,22). The relative contribution of these two factors to the increased rate of mutations at the binding sites of different TFs, and across the whole TFBSs genomic compartment in melanomas is not completely understood. To answer this question, we employed single-nucleotide-resolution CPD formation data to study the formation of the damage, and its repair, within and around 64 types of binding motifs of 48 individual TFs of 10 different families. For each TF we have identified the contribution of the DNA damage and its repair to the distribution of mutations along their binding sites. Our results reveal a complex panorama of damage formation and repair across the regions defined by the binding of the TF, which varied between families of TFs.

## MATERIALS AND METHODS

### Genomic location of transcription factor binding sites

Transcription factors ChIP-seq data were obtained from the GTRD database (23). Peak clusters called with the MACS algorithm (24) were used. These clusters are integrated by peaks of the same TF at the same genomic location identified across different experimental conditions (cell lines, treatment, etc). Thus, they represent binding sites that tend to be conserved across cell types. Next, position frequency matrices (PFM) describing the sequence of each binding motif were downloaded from the JASPAR database (25). Then, PFM were mapped within the transcription factor peaks of their corresponding TF using MOODS suite (26). DNase I hypersensitive sites (DHS) coordinates from melanocytes and skin fibroblasts were downloaded from the Epigenome Roadmap Project (27). Only those binding motifs overlapping DHS sites in both cell types were considered as active and therefore included in our analysis. The overlap between genomic locations of active motifs (±50nts from motif center) of TFs was computed using bedtools jaccard (28). Inactive transcription factor binding sites were defined as those that do not overlap DHS sites.

### Whole genome mutation data

Whole-genome somatic mutations from 183 skin cutaneous melanoma (SKCM) samples from the MELA-AU cohort (29) were retrieved. The proportion of C–T mutations was used to assess the extent of UV exposure per sample. Only the 136 samples with at least 70% of mutations being C–T were selected. Moreover, non C–T mutations were discarded.

### Whole genome CPD maps

Genome-wide distribution of CPDs immediately after UV exposure, both at normal and naked DNA was obtained from Mao *et al.* (21). CPDs distribution along the genome measured 0 and 48 h after UV exposure by Hu *et al.* (30) was obtained from Sheera Adar, one of the co-authors of the article. The authors gave the name Damage-seq to the experimental protocol developed to generate these genome-wide CPD maps. We have inherited this name in this paper to refer to the data itself. Whole-genome CPDs map in normal and naked DNA had single-nucleotide resolution and were obtained from skin fibroblast NHF1 cells.

### Expected mutation rate computation

The probability of a trinucleotide or pentanucleotide to be mutated was determined using the whole genome mutations of all 136 UV induced melanomas, normalized by the abundance of each trinucleotide or pentanucleotide in the genome. These probabilities were then used to compute the expected mutation rate of any given DNA segment, as detailed in Sabarinathan *et al.* (31). Moreover, the observed mutation rate at inactive transcription factor binding sites was also used as a background mutation rate in those analyses where we specify so.

### Repair estimation

CPD maps at different time points by Hu *et al.* (30), obtained from Sheera Adar, one of the co-authors of the article, were built by sequencing a predetermined number of reads per time-point. As a consequence, comparing CPD maps from different time-points does not inform absolute repair (or the percentage of CPDs removed after a given period of time). Instead it informs about how the distribution of CPDs changes over time by action of the repair, or in other words, how a region of the genome has been repaired with respect to another region. Thus, we called the repair inferred from comparing CPDs maps from different timepoints relative repair. In order to correct for potential experimental variations, it was computed after normalizing the number of CPDs in a given position by the total number of CPDs (from the region of interest together with the flanks or from the whole genome, as detailed below).

### Transcription factor binding site centering

For any transcription factor included in the analysis, all active binding sites along the genome were identified. Then, a region spanning 1000 nucleotides per side from the center of the binding motif was defined. The resulting 2001 bp windows were then aligned using as reference their middle position. Then, for each position, we counted the number of observed and expected mutations, and also CPDs at different timepoints after UV exposure. In those cases where the binding motif was found to be in the negative strand, the coordinates of the CPDs and mutations found in the 2001 bp window around it were inverted.

Transcription factors with fewer than 5000 active binding motifs identified in the whole genome were discarded from the analysis. Moreover, after stacking all the binding sites of a transcription factor, those with less than two mutations per position (in median) across the 2001 bp defining the analysis region were also discarded. As a result of these two filters, 328 motifs of 278 TFs are discarded from the following analyses.

When carrying out zoom-out analyses of the CPDs distribution across these 2001 bp, CPDs in each position were normalized by the total number of each CPDs in the whole 2001 bp window.

### Within motif analysis

For each specific transcription factor binding motif, only dipyrimidines conserved in at least 50% of all instances of the motif were selected to carry out the within-motif analysis of mutations, CPD formation and repair at specific dipyrimidine sites. Then, the number of mutations, CPDs at 0 and 48h after UV exposure and repair activity were assessed. Next, dipyrimidines of the same type within identical 3′ and 5′ flanking bases were sampled from the flanks of the TF (the 1500 most external bps in the 2001 bp window around the TFBS; see below). Mutations and CPDs found to overlap this set of sampled dipyrimidines were used as reference for those observed in dipyrimidines within the motif. The sampling process was performed 50 times per dipyrimidine and the results of the 50 iterations averaged.

Prior to the within motif repair analyses, 0 and 48h CPDs at each position of the motif and in the flanks were normalized by the total number of CPDs along the genome in the corresponding time point. Next, repair was computed as the percentage of CPDs decrease or increase after 48h, both in the motif and in the flanks. Finally, the difference of repair between the motif and in the flanks in percentage points was computed.

### CPDs prediction after 48 h

In order to predict the CPD distribution at the flanks and the TFBS 48h after UV exposure in the dataset from Mao *et al.* (21), we first assessed how CPDs distribution changed from 0 to 48h within Hu *et al.* (30) dataset, for each transcription factor. Then, we applied a similar transformation using Mao *et al.* (21) 0h CPDs as a starting point.

### Gene expression

The gene expression (RSEM gene-normalized) data of TCGA skin cutaneous melanoma (SKCM) cohort was obtained from the Firebrowse server (http://firebrowse.org/; TCGA data version 2016_01_28). This data was further used to check the expression level of TFs considered in this study.

### Statistics

When comparing the observed number of mutations or CPDs in a specific region with an expected distribution, being the simulated number of mutations or the CPDs in the naked DNA respectively, a chi-squared goodness of fit test was used. Alternatively, when evaluating the differences in the CPD distribution at different timepoints after UV exposure, a standard chi-squared test was employed.

## RESULTS

### Variable increase of mutation rate in the binding sites of TFs of different families

We first sought to explore the variability of mutation rate at the binding sites of different families of TFs. We collected 598,987 TFBS grouped into 64 types of binding motifs of 48 TFs from 10 different families, which are expressed across melanomas (Supplementary Figure S1), and active in melanocytes and fibroblasts (Supplementary Table S1). The binding sites were marked by TF specific ChIPseq peaks (GTRD database; (23)) which overlapped DNAse I hypersensitivity sites (DHS) detected in primary cells of both tissues (active sites). We determined the exact location of the binding motif, described by a JASPAR position weight matrix (25), within each peak using the MOODS matching algorithm (26). All analyses described below were carried out for TF binding motifs for which at least 5000 active instances were found in the genome.

**Figure 1.**
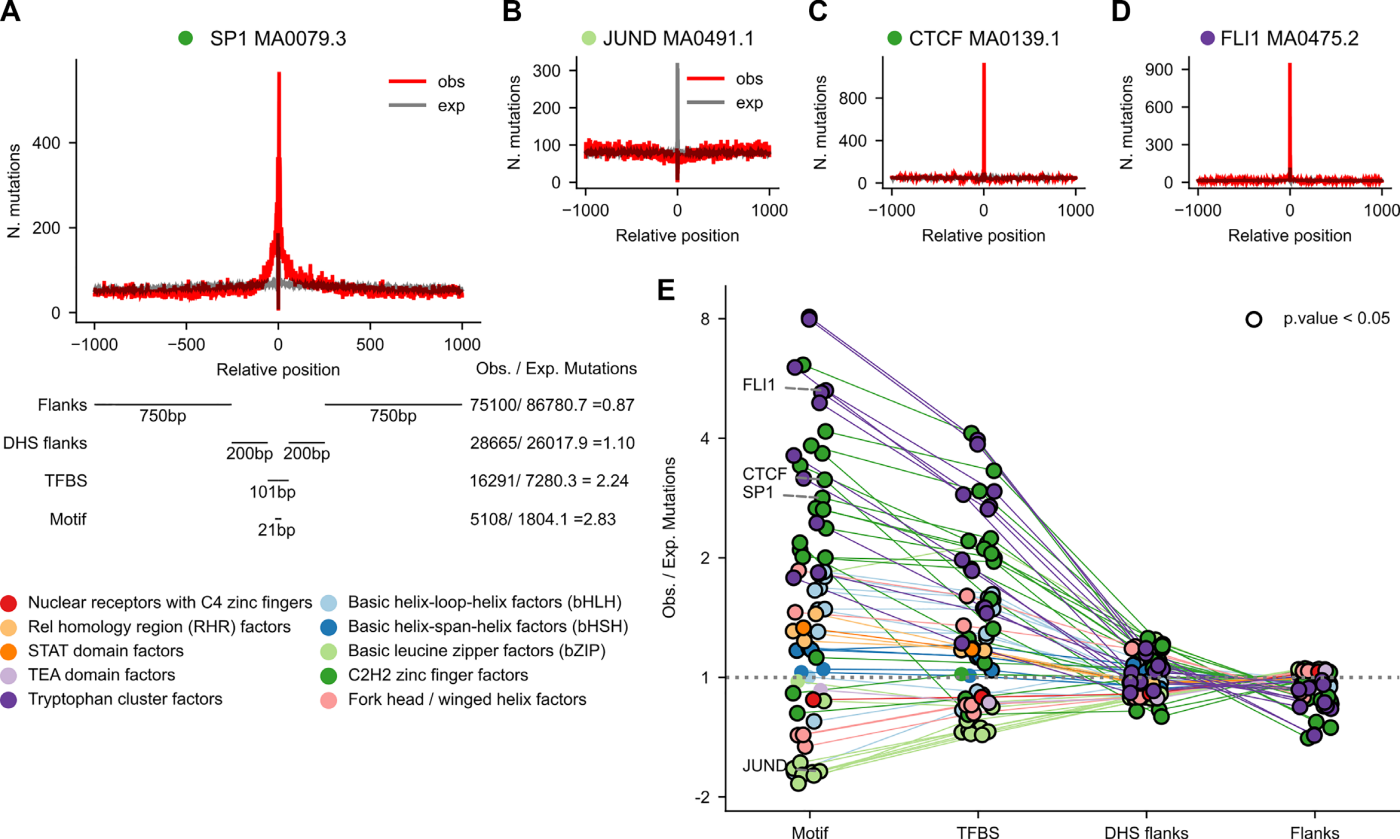
Mutation rate at TFBS in melanomas. (**A**) 2001-nucleotide sequences centered at the middle point of active TFBS are extracted from the genomic sequence. Within each sequence four areas are delimited: (from the center to the periphery) motif (21 bp), TFBS (101 bp that contain the motif), DHS flanks (400 bp), and flanks (1500 bp). The somatic mutations identified in a cohort of 136 melanomas are mapped to these sequences. Then, all 2001-nucleotide sequences containing the same type of binding motif of a TF are stacked. Mutations at each position of the stack are summed across the sequences, and the expected distribution of mutations across the 2001-nucleotide stack is computed from the profile of tri-nucleotide whole-genome substitution frequencies observed across the cohort. (**A–D**) The observed (red) and expected (gray) distributions of mutations in the stack of 2001-nucleotide sequences centered around the MA1107.1 binding motif of KLF9 (A), the MA0491.1 binding motif of JUND (B), the MA0139.1 binding motif of CTCF (C), and the MA 0475.2 binding motif of FLI1 (D). (**E**) Ratio of observed to expected mutations (in log_2_ scale) within the four regions defined across the stacks of 2001-nucleotide sequences centered across 64 types of TF binding motifs with >5000 sequences and a median number of mutations across all positions >2. Positive values correspond to higher-than-expected mutation rates at each region, whereas lower-than-expected mutation rates possess negative values. Points that correspond to instances of significant deviation from the expectation (G-test *P*-value<0.05) are encircled in black. The thin straight lines join the values computed for the regions of a given type of motif. The circles corresponding to each type of motif are colored according to the family of the corresponding TF, following the legend presented next to the panel.

In order to study the influence of the TF on the distribution of UV-induced mutations across the area around its binding site, we first defined a region of DNA spanning 1000 nucleotides at both sides of the center of the TF binding motif (Figure 1A; Supplementary Table S2). Within this 2001-nucleotide wide window, we distinguished four areas. The first region, corresponding to the binding motif itself (hereinafter motif), comprised the 21 central nucleotides in direct contact with the DNA-binding domain of the TF. Secondly, we denoted the region spanning 50 nucleotides at each side of the center of the window (including the motif) as the transcription factor binding site (TFBS). We called the region immediately flanking the TFBS, 200 nucleotides on each side, DHS flanks. DHS flanks are expected to consist mostly of protein-free DNA. The remaining 1500 bp outside the DHS flanks were labeled flanks. These four regions were thus defined in each of the 598,987 collected active TFBS. Overlapping binding motifs (generally of the same family) appear within the 2001-nucleotide sequence of certain TFs (Supplementary Figure S2A), and the binding sites of some TFs appear closer than others—on average—to the TSS of genes (Supplementary Figure S2B).

From a cohort of 136 UV-exposed melanomas (29) we obtained the mutations overlapping these 598,987 2001-nucleotide wide regions. The sequences corresponding to the binding sites of the same type of motif of a given TF (Supplementary Table S1) were stacked, and the mutations observed in each of the 2001 columns of the resulting matrix summed (red line in Figure 1A). We derived an expected mutational frequency at each position of the stacked sequences by randomizing the mutations observed in each of them following the tri-nucleotide substitution frequencies (black line in Figure 1A; penta-nucleotide frequencies, Supplementary Figure S3A) computed using all mutations in the whole genomes of the 136 melanomas. An alternative expected mutational frequency was derived from mutations observed at inactive TFBS (i.e., those not overlapping DHS and thus expected to be TF-free) with very similar results (Supplementary Figure S3B).

Figures 1A–D present the observed and expected mutation rates across the stacked 2001-nucleotide sequences of four binding motifs of TFs SP1, JUND, CTCF and FLI1 (all TF motifs analyzed appear in Supplementary Figure S4, and results are summarized in Table 1). They represent a variety of patterns of mutation rate, including broad (extending beyond the motif) and narrow higher-than-expected mutation rate, as well as lower-than-expected mutation rate (e.g., the binding motif of JUND).

**Table 1.** Results of all analyses across motifs of different TFs. The number of motifs with significant increase or decrease of the rate of observed mutations, CPDs (at 0h), or repair with respect to the expectation are presented

			Mutation rate		Damage rate (CPDs at 0h)		Repair rate (with respect to DHS flanks)		Repair rate (with respect to flanks)	
Family	TFs	motifs	Significant increase	Significant decrease	Significant increase	Significant decrease	Significant increase	Significant decrease	Significant increase	Significant decrease
Basic helix-loop-helix (bHLH)	7	10	7	2	1	8	0	8	2	6
Basic helix-span-helix (bHSH)	2	5	3	0	0	5	0	5	0	5
Basic leucine zipper (bZIP)^a^	10	10	1	8	0	10	0	4	9	1
C2H2 zinc finger	14	17	15	2	1	11	1	14	0	13
Fork head/winged helix	3	5	2	3	0	0	3	2	3	2
Nuclear receptors with C4 zinc fingers	1	1	0	1	0	0	0	1	0	1
Rel homology region (RHR)	2	3	3	0	0	3	0	2	0	2
STAT domain	1	1	1	0	0	0	1	0	1	0
TEA domain	1	1	0	0	0	0	0	0	1	0
Tryptophan cluster^b^	7	11	11	0	10	0	5	1	6	2

^a^TF Family showing significant decrease of mutation rate within the TFBS.

^b^TF Family showing significant increase of CPD formation rate within the TFBS, and significant increase of the rate of CPD repair.

The behavior of the represented motifs of these four TFs is paradigmatic of their corresponding families (SP1 and CTCF, C2H2 zinc fingers; JUND, basic leucine zipper; FLI1, tryptophan cluster; Figure 1E, Supplementary Figure S3A, B). A higher-than-expected (positive log_2_ fold-change in Figure 1E) mutation rate is observed across the motif of TFs of most families. The mutation rate of the motifs of most basic leucine zipper and fork head TFs, on the other hand, is significantly lower than expected. In most instances, the excess of the mutation rate over the expected value in the TFBS is smaller than that observed at the motif, i.e. producing a narrow peak. In some TFs, notably SP1, this reduction is less abrupt, yielding rather broad peaks of observed mutations. It is possible, to some extent, that broader peaks correspond to binding sites close to those of other TFs, or more generally, close to the TSS where the transcription machinery sits (Supplementary Figures S2A, B). This could explain, for example, the case of SP1, the binding sites of which tend to co-occur in the same regulatory sequences as those of other TFs of the Tryptophan cluster family. The increase or decrease of mutation rate in DHS flanks is more modest that that observed in TF motifs. The increase of observed mutation rate (with respect to the expectation) tends to be more apparent at the motifs of TFs with higher expression—a proxy of higher occupancy of their binding sites—across melanomas (Supplementary Figure S5).

**Figure 2.**
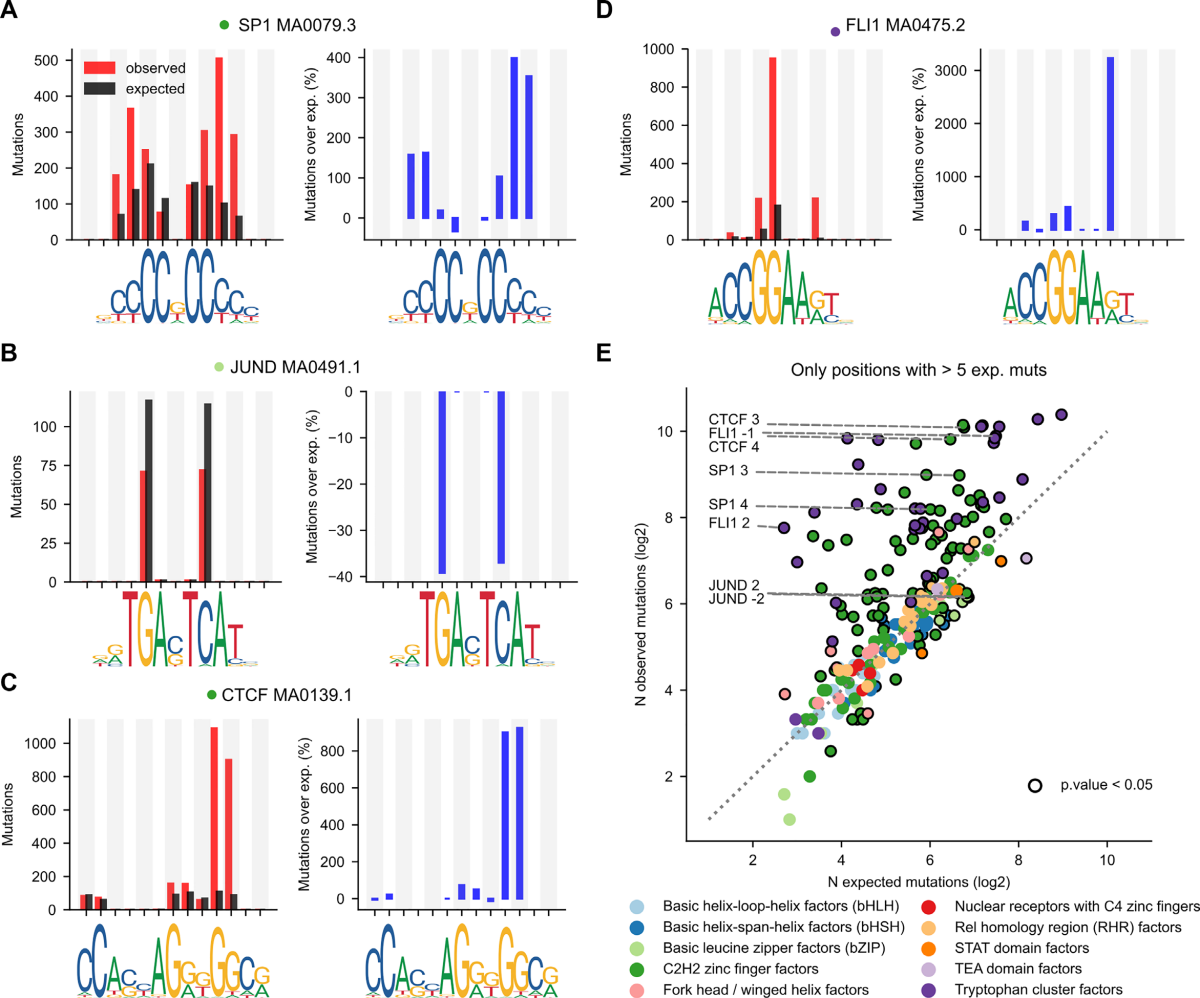
Mutation rate at specific positions within the TF binding motif. (**A–D**) The left graph in each panel presents the observed (red bar) and expected (black bar) number of mutations at each mutated dipyrimidine position of the four binding motifs shown in Figures 1A–D. To compute the expected mutations at each dipyrimidine position the tetramer containing the dipyrimidine was sampled from the flanks of the same 2001-nucleotides sequence, and their observed number of mutations averaged. The right graph presents the corresponding percentage of increase of the rate of mutations over that expected (blue bar) at each mutated dipyrimidine position. Positive values thus correspond to increased mutation rate, while negative values occur at positions with decreased mutation rate. (**E**) Scatterplot representing the relationship between the number of observed and expected mutations (in log_2_ scale) at all dipyrimidine positions within all binding motifs included in the study. Each dot, hence corresponds to an individual dipyrimidine position in a binding motif colored following the family of the corresponding TF, according to the legend below the panel. Dipyrimidine positions with significant increased or decreased number of mutations with respect to the expectation (G-test *P*-value < 0.05) are encircled in black.

We next zoomed-in on the motif area to explore whether the deviations of the mutation rate from the expectations are roughly distributed across dipyrimidine sites or driven by just one or few of them, as recently observed for the ETS1 binding sites (21,22). We focused on dipyirimidines that are present in at least 50% of the instances of a binding motif (Figure 2). The number of mutations observed in either base of the dipyrimidine were summed and compared to their expected numbers, based on random samples of the same tetranucleotides (the dipyrimidine and its immediate flanks) from the flanks of the corresponding 2001-nucleotide sequence (Materials and Methods). Finally, we subtracted the expected rate of mutations at each position from their observed number, and made the difference relative to the expected (percent of mutations relative to expected).

At some TF binding motifs –as previously observed for ETS1—the increase in the number of UV-induced mutations with respect to the expectation is mainly driven by one or few dipyrimidines (Figure 2A–D; Supplementary Figure S6 and Supplementary Table S2). Specifically, some dipyrimidine sites within Tryptophan Cluster and C2H2 Zinc Fingers TF motifs tend to bear more mutations than expected (Figure 2E). At these sites features other than sequence context influence the rate of mutations, pointing at the influence of bound TFs on the formation of UV-induced photoproducts and/or their repair (21). In most of the motifs of TFs of other families, the number of observed mutations at dipyrimidine sites does not differ from that expected from their sequence context.

In summary, as previously observed (19–22) there is a general increase of the mutation rate at the sites of bound TFs. Interestingly, at the binding sites of some TFs there is a noticeable decrease of the mutation rate. The extent and direction of variation in mutation rate depends on the family of TFs. In some families the change in mutation rate is not limited to the binding motif, but extends to the entire TFBS.

### Increased CPDs formation within TFBS of tryptophan cluster TFs

**Figure 3.**
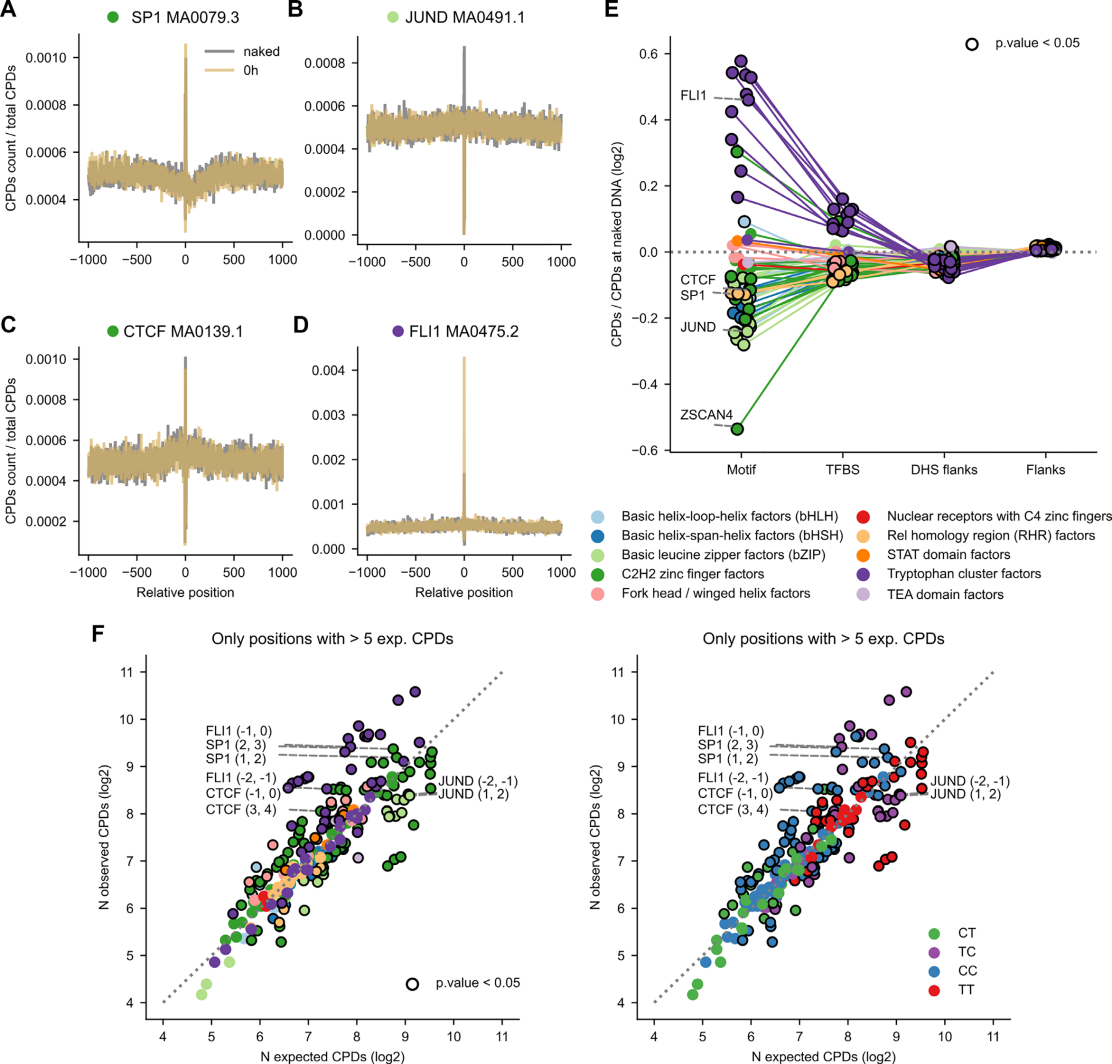
CPD formation rate at TFBS. (**A–D**) Distribution of the observed (amber) or expected (dark gray) rate of CPDs formed upon UV irradiation within 2001-nucleotides sequences centered at the middle position of the TFBS shown in Figures 1 and 2. (**E**) Ratio of observed to expected CPDs formation rates (in log_2_ scale) within the four regions studied. Positive values correspond to higher-than-expected CPD formation rates at each region, whereas lower-than-expected CPD formation rates possess negative values. Points representing instances with significant deviations from the expectation (G-test *P*-value < 0.05) are encircled in black. Thin straight lines join the values computed for the regions of a given type of motif. (**F**) Scatterplots representing the relationship between the number (log_2_) of observed and expected CPDs formed at specific dipyrimidine positions within each type of motif. In the left plot, the dots representing the motifs are colored following TF families, while in the right plot, their colors correspond to the type of dipyrimidine where they occur.

Deviations of the mutation rate at the binding sites of specific TFs with respect to the expectation may be driven by the influence of the protein on the formation of UV-induced lesions (21,22) and/or on the efficiency of the repair of these lesions by NER (19,20). Thus, we next analyzed the rate of formation of CPDs in TFBS across TF families. To this end, we employed nucleotide-resolution CPD formation data generated by Mao *et al.* on irradiated fibroblasts (21). As with mutations, we mapped CPDs (Supplementary Figure S7A) across the 2001-nucleotide wide sequences in the stack of each TF motif (amber line in Figure 3A). CPDs generated in the naked DNA of each binding site in a parallel experiment were used to compute the expected rate of DNA damage across each TF motif (dark gray line in Figure 3A). An analysis of the rate of CPD formation across the same set of TFBS employing an independent whole-genome UV-damage map showed coherent results for several TF families and differences across others (Supplementary Figure S7B). To correct for experimental variability, the observed and expected CPDs at each individual position were normalized by the total number of observed CPDs across the whole 2001-nucleotide window in either experiment.

Figures 3A–D (Supplementary Figure S8 and Supplementary Table S2) present the distribution of CPD formation rate across the binding motifs of TFs of different families (summarized in Table 1). While higher-than-expected rates of CPDs appear within the binding motif of FLI1, in the other three cases illustrated in Figures 3A–D, the rate of CPDs formed when the binding site is occupied is lower than that of naked DNA. Indeed, as a rule TFs of the Tryptophan Cluster family exhibit significantly higher-than-expected CPD formation within their binding motifs (Figure 3E). This is probably attributable to the influence of the TF bound to the motif, as has been shown for ETS1 (21). On the contrary, TFs of other families show lower-than-expected (with many significant instances) CPD formation within the motif. This suggests that in some cases the bound TF could have a protective effect on its binding sites.

Zooming-in on specific dipyrimidines within the motif (following the same approach as with mutations; Supplementary Figure S9 and Supplementary Table S2), the deviations from the expected rate of CPDs was less widespread than in the case of the mutation rate. Only a few motifs presented large higher- (some Tryptophan Cluster TFs) or lower-than-expected (some C2H2 Zinc Finger and Basic Leucine Zipper TFs) rates of CPD formation. No clear pattern of deviation of CPD formation from the expectation across motifs were observed across the four dipyrimidine types (Figure 3F).

Summing up, CPD formation rate is higher than expected in the motifs of TFs of the Tryptophan Cluster family, which could contribute to their higher-than-expected rate of mutations. However, for the majority of TF motifs analyzed, the rate of CPD formation is not above the expectation and thus, it does not explain their increased mutation rate.

### Widespread decrease of CPD repair within TFBS

The distribution of mutations across a genomic area is determined by the interplay between the distribution of DNA lesions left by a damaging agent and the efficiency of their repair. We thus next focused on the efficiency of repair of CPDs within TFBS across TF families. Previous studies have shown that the efficiency of NER in the repair of CPDs within the TFBS is diminished with respect to that at its flanks, which has been attributed to reduced accessibility to the lesions due to the bound TF (19,20).

**Figure 4.**
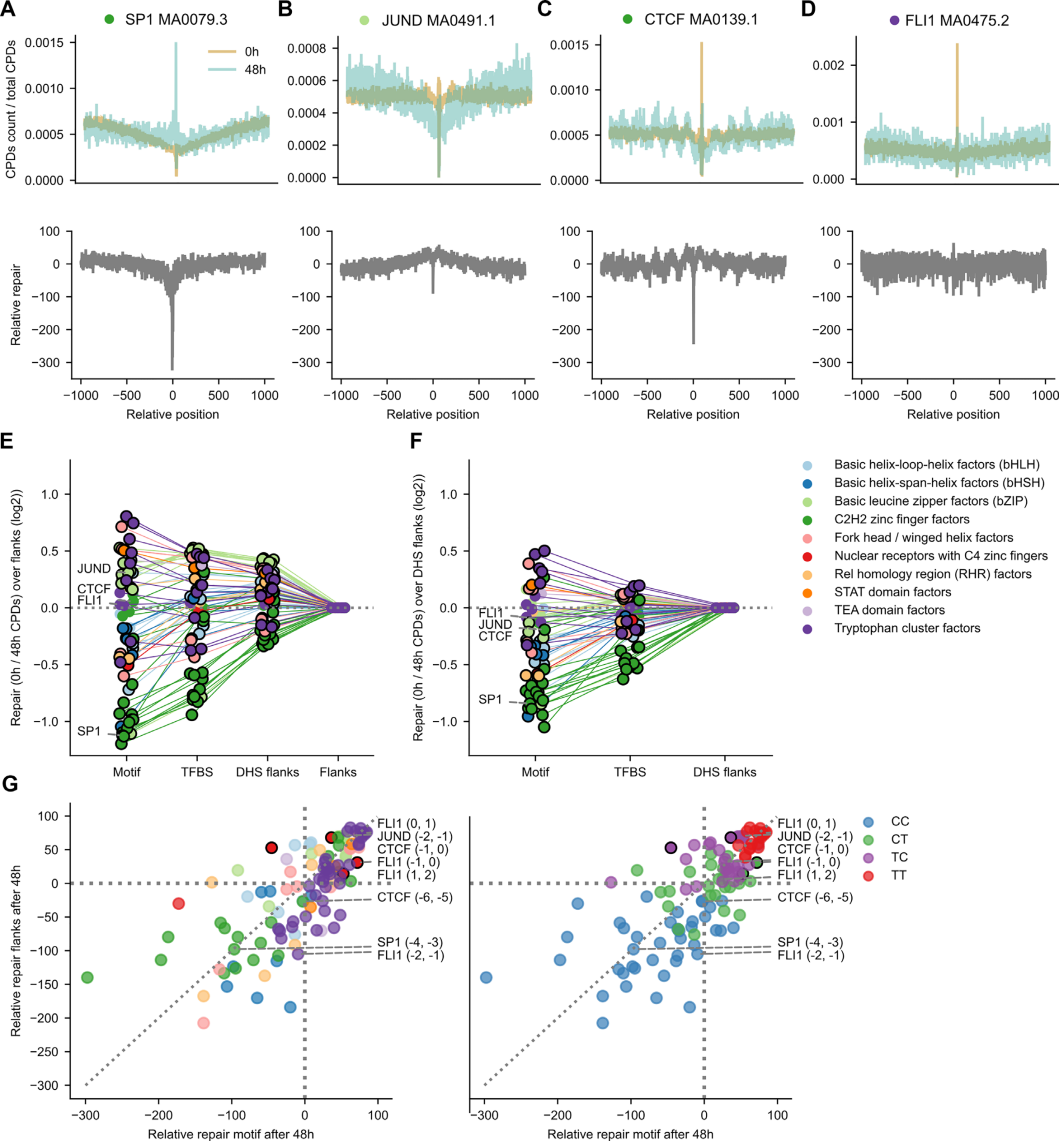
Relative repair of CPDs at TFBS. (**A–D**) Relative activity of repair of CPDs in the four TF binding motifs presented in previous Figures. Top panels represent the rate of CPDs experimentally generated immediately after irradiation (0h, ambar) and the rate of CPDs persisting 48 h (blue) after irradiation. (Both CPD rates are computed relative to the number of CPDs across the entire TFBS.) The subtraction of the latter from the former, nucleotide-by-nucleotide yields the relative rate of repair across each type of binding motif. (**E**) Ratio of the relative repair rates of CPDs at motifs, TFBS or DHS flanks with respect to flanks (in log_2_ scale). Positive values correspond to relative CPD repair rates that are larger at a given region than at the flanks, whereas lower-than-flanks mutation ratios possess negative values. Points encircled in black represent instances of significant deviation from the expectation (G-test *P*-value < 0.05). Thin straight lines join the values computed for the regions of a given type of motif. The points representing the ratios computed for each region of each type of motif are colored according to the family of the corresponding TF, following the legend presented next to the panel. (**F**) Ratio of the relative repair rates of CPDs at motifs or TFBS with respect to DHS flanks (in log_2_ scale). Notation as in panel (E). (**G**) Scatterplots representing the relationship between the percent of repaired CPDs at specific dipyrimidine positions within individual motifs (after 48 h) and the average repair rate for the same type of dipyrimidine in the same context (tetranucleotide) within the flanks. Each dot corresponds to an individual dipyrimidine. Dots above the diagonal represent dipyrimidines repaired at a higher rate than dipyrimidines in the same context in the flanks. On the other hand, dots below the diagonal correspond to dipyrimidines at positions that experience lower relative repair rate.

To determine the efficiency of NER across the binding sites of the TFs under study, we exploited nucleotide-resolution CPDs mapped by Hu *et al.* immediately after irradiation of cells (0h) and 48h after UV exposure (30). We mapped these CPDs to the 2001-nucleotide wide sequences containing the TF binding motifs. The number of CPDs at each position per time point was normalized by the total number of CPDs observed within the window (Materials and Methods). Then, the activity of repair was computed as the percentage of normalized CPDs repaired 48h after exposure (blue line in top panel of Figure 4A-D) with respect to the normalized number recorded immediately after irradiation (amber line in top panel of Figure 4A-D), and is represented as a gray line at the bottom panel of Figure 4A-D. In the three first motifs represented in Figures 4A–D (Supplementary Table S2), the fraction of repaired CPDs at the center of the motif is smaller than at the flanks. The exception is the motif of FLI1 (Figure 4D), with slightly higher repair rate within the motif than at the flanks. Supplementary Figure S10 presents the repair efficiency across all analyzed TF motifs. Results are summarized in Table 1.

Taking the flanks as reference of repair activity (Figure 4E) we are able to obtain an overall view of the efficiency of repair in different regions of the binding sites across TFs. In most cases, the motif is repaired at significantly lower rates than the flanks, with several Tryptophan Cluster and Basic Leucine Zipper TFs as exceptions. If the DHS flanks—largely protein-free DNA—are taken as reference (Figure 4F), the widespread decrease of repair activity across motifs and TFBS becomes more apparent. This supports the hypothesis that the decrease in repair efficiency observed at the majority of TFBS is caused by the impairment in NER accessibility to the damage at the motif and the TFBS (19,20).

A zoomed-in analysis of the repair of CPDs at specific dipyrimidine positions within motifs (Figure 4G, Supplementary Figure S11 and Supplementary Table S2) reveals that CPDs within binding motifs tend to be repaired at the same rate as their counterparts within the binding site, with some variability among families. Intriguingly, we observed that CPDs involving CC dipyrimidines tend to occupy lowly-repaired positions (Figure 4G), while those of TT or TC dipyrimidines were at highly repaired positions. This is true along the whole genomic sequence, and is therefore not caused by the binding of proteins to the DNA (Supplementary Figure S12). This observation suggests that different dipyrimidines—possible by virtue of differences in the torsion of the B-DNA helix—are repaired at intrinsically different rates.

The decreased CPDs repair within TF motifs is, in conclusion, widespread across TFs, regardless of their family, with some exceptions.

### The generation of UV-induced mutations within TFBS

**Figure 5.**
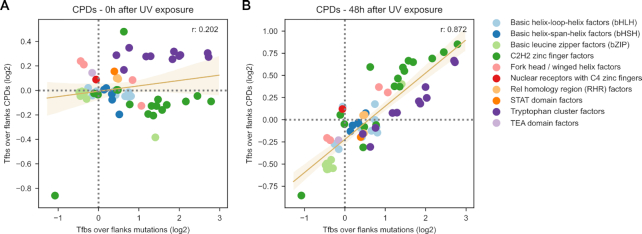
The UV mutational process in TFBS. (**A**) Relationship between the ratio (log_2_) of TFBS-to-flanks CPDs (y-axis) computed immediately after irradiation and the ratio (log_2_) of TFBS-to-flanks mutations (x-axis). (**B**) Relationship between the ratio (log_2_) of TFBS-to-flanks CPDs (y-axis) computed 48 h after irradiation and the ratio (log_2_) of TFBS-to-flanks mutations (x-axis). Dots represent specific motifs. The trendline and the Pearson's correlation coefficients computed for both relationships are presented in the graph.

On the basis of the results of the analyses outlined in the two previous sections, we hypothesized that the rate of CPDs remaining at 48h across TFBS would be a good predictor of the rate of mutations observed at each of them. This hypothesis is based on the assumption that the rate of mutations across TFBS is ultimately shaped by the rate of CPDs generated by UV within each TFBS and the efficiency of NER correcting them. We estimated the number of CPDs at 48h using inferred repair activity rather than directly using CPDs mapped in the DamageSeq experiment (Materials and Methods). In agreement with our hypothesis, the rate of CPDs at 48h correlated better with the mutation rate of the motif relative to the flanks than that observed at 0h (Figure 5A, B and Supplementary Figure S13). This supports the notion that a general impairment of NER caused by the protein bound to the DNA drives the observed increase of mutation rate across most TFBS.

## DISCUSSION

The observation that the burden of mutations at promoter regions—and in particular in TFBS—in melanomas was unexpectedly high paved the way for the dissection of the detailed influence of the bound TF on the formation of UV-induced lesions (21,22) and their repair (19,20). Different studies discovered that TFs bound may increase the rate of CPD formation at certain positions of their binding motifs (21,22) and that they interfere with the accessibility of the NER machinery to CPDs, thus hindering their repair at TFBS (19,20). The opportunity to carry out this careful dissection emerged in recent years owing to the ability to map UV-induced damage (21) and its repair (30) at single-nucleotide resolution, and the availability of the whole-genome sequence of UV-exposed skin tumors (29). The main motivation of the present work consisted in unraveling the specific contribution of these two factors (UV-induced damage and its repair) to the abnormally high burden of UV-induced mutations at TFBS for TFs of different families. The results of these analyses—supporting general observations discussed below, but also cases that show different behavior—across different TF families are summarized in Table 1.

Although we undertook this work with a comprehensive toolbox of maps of UV-induced damage, repair, and mutations, we must first acknowledge its intrinsic limitations. Using antibodies-based experiments in which CPDs are mapped across the genome at different times after irradiation we are able to infer the rate of repair at TFBS, but the maps may be biased against the recognition of certain types of CPDs (32) (Supplementary Figure S7A). On the other hand, whole-genome maps of CPDs computed through a potentially less biased enzyme-based assay are available only immediately after irradiation. We therefore inferred the repair intensity through the comparison of CPDs mapped at two time points through the antibodies-based assay. This approach –unlike the direct mapping of the repair (XR-seq) allowed us to correct for the rate of the damage immediately after irradiation. This should result in more accurate estimates of repair intensity irrespective of the rate of CPDs generated by the exposure. In XR-seq segments, moreover, the exact location of the CPD is difficult to map.

Due to the possible aforementioned bias, we also indirectly estimate the number of CPDs at different areas of individual TFBS 48h after irradiation on the basis of their original numbers estimated through the enzymatic experiment, and the previously inferred repair rate. Whole-genome mapping of CPDs at late time points after irradiation using less biased experiments will support the direct calculation of CPD rates at different TFBS. A second limitation of the analysis stems from the fact that while CPDs have been mapped in a fibroblast cell line (21,30), the source of mutations are melanomas (29), which derive from melanocytes, a different cell type. We addressed this hurdle by restricting the analysis to TFBS active in both cell types (27). These caveats aside, the estimated rate of CPDs after 48 h is accurate enough to show a high correlation (*r* = 0.872; Figure 5B) with the mutation rate at the same sites.

Our results show that the UV mutational process in the TFBS—i.e. the distribution of mutations across different areas that results from the interplay between the formation of the CPDs and their repair—is complex, and varies widely between families of TFs. First, we ratified our previous observation (19) that the higher-than-expected mutation rate is widespread in melanomas across the binding motifs of TFs. However, we also determined that the binding sites of some TFs, such as Basic Leucine Zipper TFs actually exhibit lower-than-expected mutation rate. In the majority of TFs with increased mutation rate, the causes are complex and diverse. The deviation from the B-DNA structure that TFs of the Tryptophan Cluster family (such as ETS1 (21,22)) force on their binding motif results in higher-than-expected rate of CPDs. This is probably the main driver of the unexpected mutational peak observed in melanomas in the binding sites of these TFs, reported elsewhere (21,22). In the TFs of this family, the mutational peak that arises from the increased CPD formation tends to be confined to one or few dipyrimidines within the motif that contacts the protein. We also found that the decreased accessibility of NER that results in a reduced activity of repair of UV-induced lesions is observed across the binding sites of TFs of most families and is thus a more widespread cause of their increased mutation rate. Some TFs exhibit broader peaks of mutations at their binding sites than others. This could be explained by multiple TFs that bind cooperatively, or that share the same or overlapping motifs or, more generally, that bind close to the TSS where the transcription machinery sits (Supplementary Figure S2A,B) (33).

It is important to note that this interpretation of the observed decrease in repair efficiency assumes that TFs –for the most part– remain bound to their sites after CPDs formation. At least two pieces of evidence support this assumption. On the one hand, the higher the affinity of the TF for its binding sites, the more impaired their repair (19), on the other, highly expressed genes—i.e. those with higher occupancy of TFBS—exhibit higher mutational peaks at their promoter regions (20). This raises the intriguing possibility that the significant increase in the rate of DNA repair registered at the binding sites of Tryptophan cluster TFs—an exception in this regard (Table 1)—is caused by the TF vacating the site, due to the distortion of the DNA double helix.

The study presented here also introduces some conceptual and methodological advances that may be applicable to the analysis of the interaction between mutational processes and features of the chromatin. Using the amount of DNA damage observed in a genomic region (TFBS in this case) and the estimated rate of repair during 48h, we predict the rate of damage that remains at that time. This idea could be applied to the estimation of other types of DNA damage across other genomic features. Furthermore, through the correlation between the damage at earlier and later time points and the mutation rate, we demonstrated the role of repair in the formation of mutations.

Finally, even though focused on TFBS, our work is an example of how the path leading from the creation of DNA lesions to mutations can be reconstructed and studied in a stepwise manner. Therefore, we believe a similar approach could be used to understand other mutational biases observed along the genome, either caused by UV or any other mutational processes.

## Supplementary Material

gkaa1219_Supplemental_Files
