# Supplementary material for: Variable interplay of UV-induced DNA damage and repair at transcription factor binding sites
Source: Nucleic Acids Res. 2020 Dec 21;49(2):891–901. doi: 10.1093/nar/gkaa1219 (PMC7826277; doi:10.1093/nar/gkaa1219)
Supplement: gkaa1219_Supplemental_Files [file gkaa1219_supplemental_files.zip › Supplementary_Figures.pdf]

Supplementary Figures

Figure S1

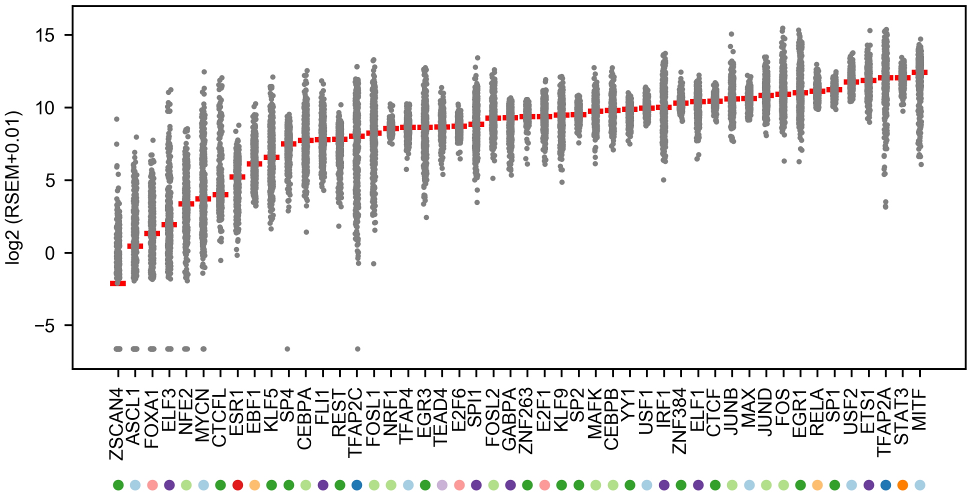

log2 (RSEM+0.01)
15
10
5
0
−5
ZSCAN4
ASCL1
FOXA1
ELF3
NFE2
MYCN
CTCFL
ESR1
EBF1
KLF5
SP4
CEBPA
FLI1
REST
TFAP2C
FOSL1
NRF1
TFAP4
EGR3
TEAD4
E2F6
SPI1
FOSL2
GABPA
ZNF263
E2F1
KLF9
SP2
MAFK
CEBPB
YY1
USF1
IRF1
ZNF384
ELF1
CTCF
JUNB
MAX
JUND
FOS
EGR1
RELA
SP1
USF2
ETS1
TFAP2A
STAT3
MITF

Figure S1. Expression of TFs in skin cancer samples across TCGA cohort.
Each dot represents a sample, and the red horizontal line indicates the median expression value of each TF. TFs are labeled following the family color legend introduced in Figure 1 (dots below the x axis).

Figure S2

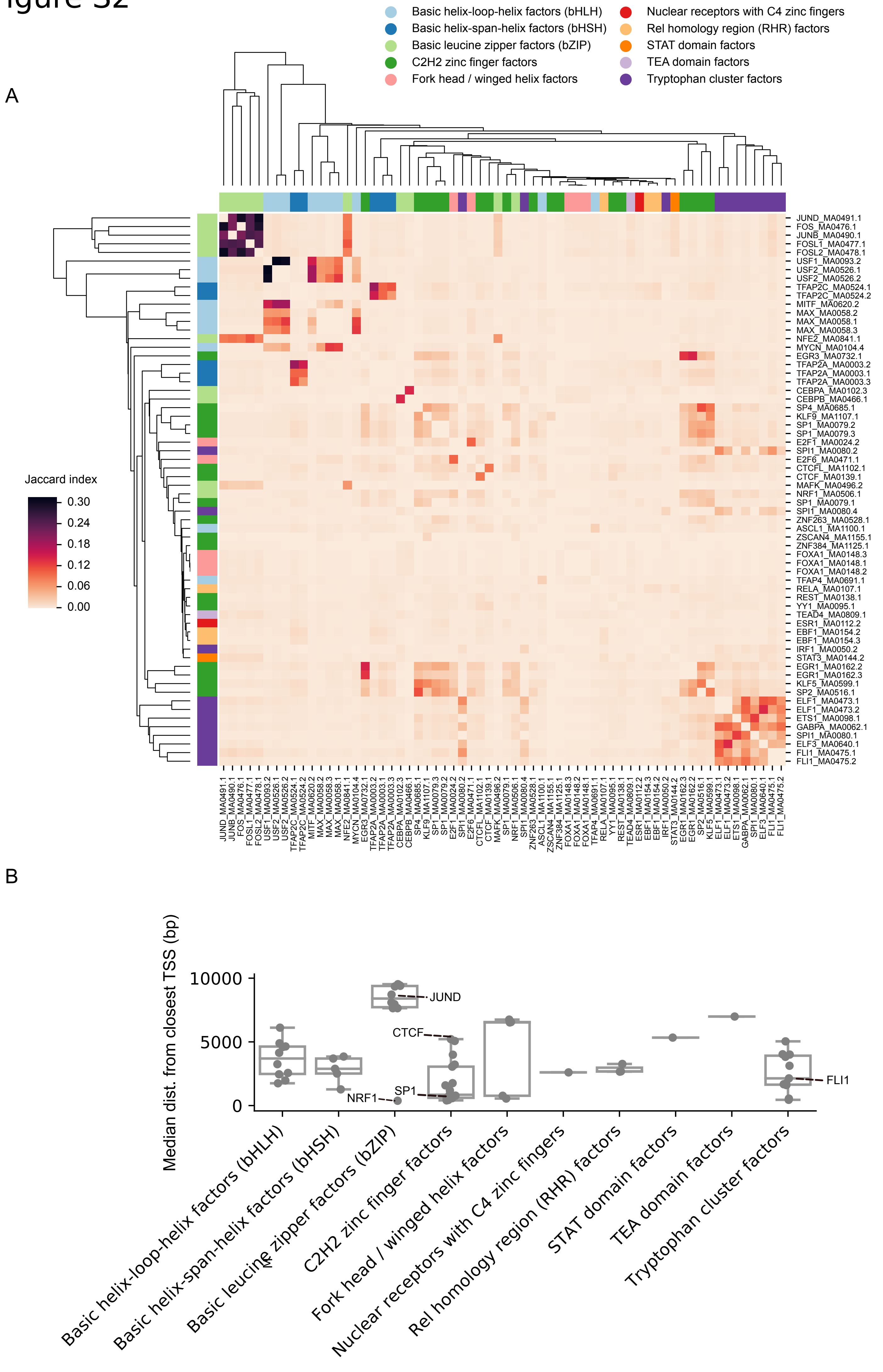
A
B
Basic helix-loop-helix factors (bHLH)
Basic helix-span-helix factors (bHSH)
Basic leucine zipper factors (bZIP)
C2H2 zinc finger factors
Fork head / winged helix factors
Nuclear receptors with C4 zinc fingers
Rel homology region (RHR) factors
STAT domain factors
TEA domain factors
Tryptophan cluster factors
Jaccard index
0.30
0.24
0.18
0.12
0.06
0.00
JUND_MA0491.1
FOS_MA0476.1
JUNB_MA0490.1
FOSL1_MA0477.1
FOSL2_MA0478.1
USF1_MA0093.2
USF2_MA0526.1
USF2_MA0526.2
TFAP2C_MA0524.1
TFAP2C_MA0524.2
MITF_MA0620.2
MAX_MA0058.2
MAX_MA0058.1
MAX_MA0058.3
NFE2_MA0841.1
MYCN_MA0104.4
EGR3_MA0732.1
TFAP2A_MA0003.2
TFAP2A_MA0003.1
TFAP2A_MA0003.3
CEBPA_MA0102.3
CEBPB_MA0466.1
SP4_MA0685.1
KLF9_MA1107.1
SP1_MA0079.2
SP1_MA0079.3
E2F1_MA0024.2
SPI1_MA0080.2
E2F6_MA0471.1
CTCFL_MA1102.1
CTCF_MA0139.1
MAFK_MA0496.2
NRF1_MA0506.1
SP1_MA0079.1
SPI1_MA0080.4
ZNF263_MA0528.1
ASCL1_MA1100.1
ZSCAN4_MA1155.1
ZNF384_MA1125.1
FOXA1_MA0148.3
FOXA1_MA0148.1
FOXA1_MA0148.2
TFAP4_MA0691.1
RELA_MA0107.1
REST_MA0138.1
YY1_MA0095.1
TEAD4_MA0809.1
ESR1_MA0112.2
EBF1_MA0154.2
EBF1_MA0154.3
IRF1_MA0050.2
STAT3_MA0144.2
EGR1_MA0162.2
EGR1_MA0162.3
KLF5_MA0599.1
SP2_MA0516.1
ELF1_MA0473.1
ELF1_MA0473.2
ETS1_MA0098.1
GABPA_MA0062.1
SPI1_MA0080.1
ELF3_MA0640.1
FLI1_MA0475.1
FLI1_MA0475.2
Median dist. from closest TSS (bp)
10000
5000
0
JUND
CTCF
SP1
NRF1
FLI1
Basic helix-loop-helix factors (bHLH)
Basic helix-span-helix factors (bHSH)
Basic leucine zipper factors (bZIP)
C2H2 zinc finger factors
Fork head / winged helix factors
Nuclear receptors with C4 zinc fingers
Rel homology region (RHR) factors
STAT domain factors
TEA domain factors
Tryptophan cluster factors

Figure S2. Overlap of motifs with other DNA-bound proteins.

(A) Overlap of motifs of pairs of TFs. The overlap of the motifs bound by each pair of TFs is measured through the Jaccard index, considering motifs that overlap at least 1 nucleotide as overlapping. A Jaccard Index close to 1 would indicate overlap of all motifs of both members of the pair.

(B) Distribution of the average distance between motifs of TFs of different families and the closest Transcription Start Site. The names of exemplary TFs (represented across main Figures) are highlighted. The motifs of some instances of TFs with broad mutational peaks (such as SP1 and NRF1; see Fig. S4) do appear closer to the TSS than others.

Figure S3

A

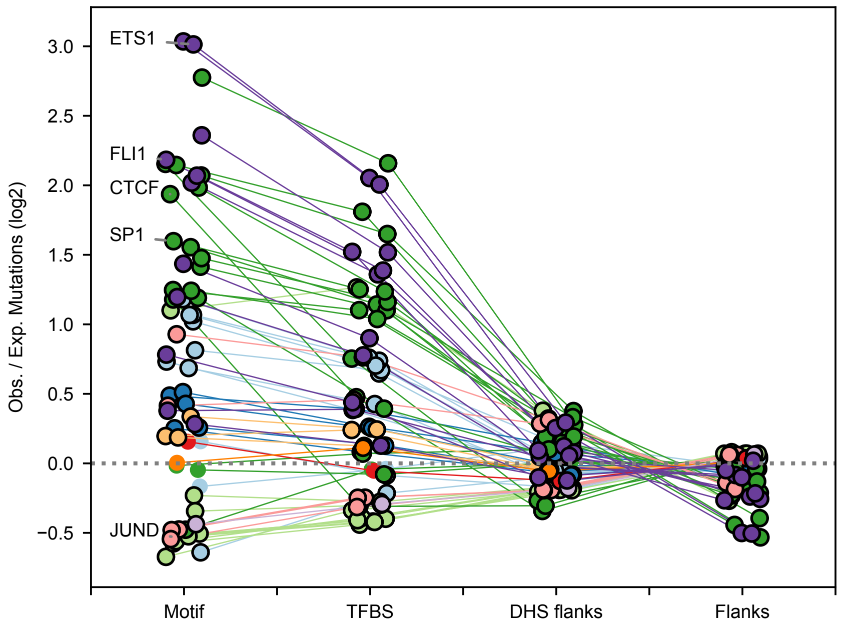
ETS1
FLI1
CTCF
SP1
JUND
Obs. / Exp. Mutations (log2)
3.0
2.5
2.0
1.5
1.0
0.5
0.0
−0.5
Motif
TFBS
DHS flanks
Flanks

B

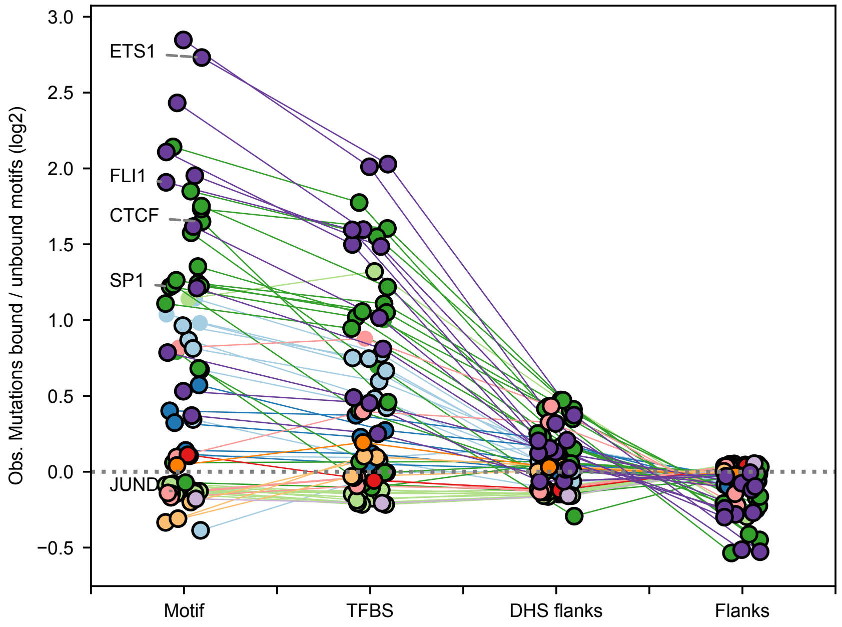
ETS1
FLI1
CTCF
SP1
JUND
Obs. Mutations bound / unbound motifs (log2)
3.0
2.5
2.0
1.5
1.0
0.5
0.0
−0.5
Motif
TFBS
DHS flanks
Flanks

Figure S3. The increased rate of mutations at TFBS measured using different backgrounds

(A) Ratio of observed to expected mutations (in log2 scale) within the four regions defined across the stacks of 2001-nucleotide sequences centered across 64 types of TF binding motifs. This is equivalent to the graph presented in Figure 1e, but the expected mutation rates have been computed based on the frequencies of pentameric (rather than trimeric) nucleotide change sequence contexts.

(B) Ratio of observed mutations in TF bound binding motifs to observed mutations in binding sites that are inactive (in log2 scale) within the four regions defined across the stacks of 2001-nucleotide sequences centered across 64 types of TF binding motifs (see Methods for details). This is equivalent to the graph presented in Figure 1e, but the observed mutation rate in binding motifs not overlapping a DHS site have been used as reference (rather than computing an expected mutation rate).

Figure S4

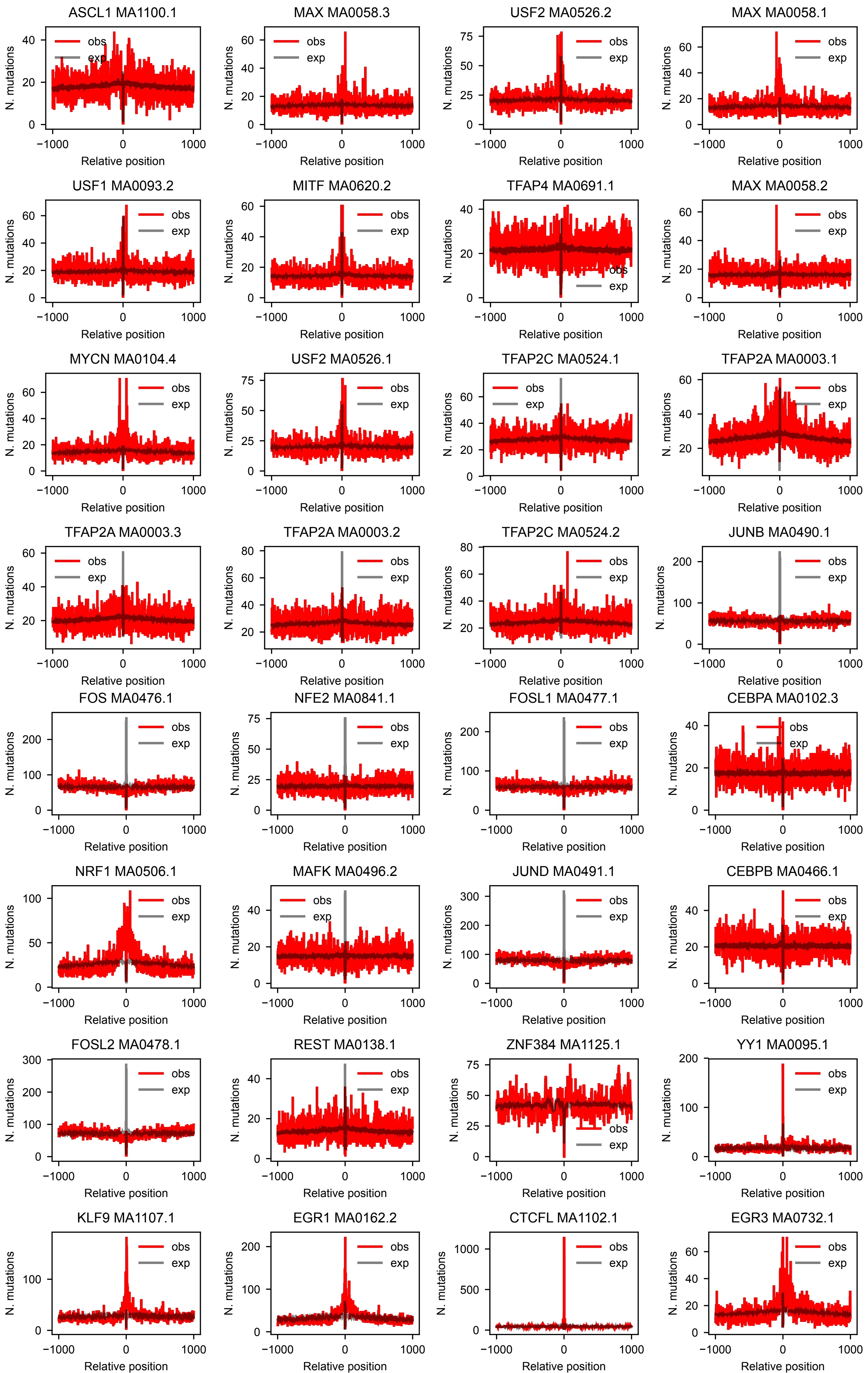
ASCL1 MA1100.1
MAX MA0058.3
USF2 MA0526.2
MAX MA0058.1
USF1 MA0093.2
MITF MA0620.2
TFAP4 MA0691.1
MAX MA0058.2
MYCN MA0104.4
USF2 MA0526.1
TFAP2C MA0524.1
TFAP2A MA0003.1
TFAP2A MA0003.3
TFAP2A MA0003.2
TFAP2C MA0524.2
JUNB MA0490.1
FOS MA0476.1
NFE2 MA0841.1
FOSL1 MA0477.1
CEBPA MA0102.3
NRF1 MA0506.1
MAFK MA0496.2
JUND MA0491.1
CEBPB MA0466.1
FOSL2 MA0478.1
REST MA0138.1
ZNF384 MA1125.1
YY1 MA0095.1
KLF9 MA1107.1
EGR1 MA0162.2
CTCFL MA1102.1
EGR3 MA0732.1
obs
exp
N. mutations
Relative position
−1000
0
1000

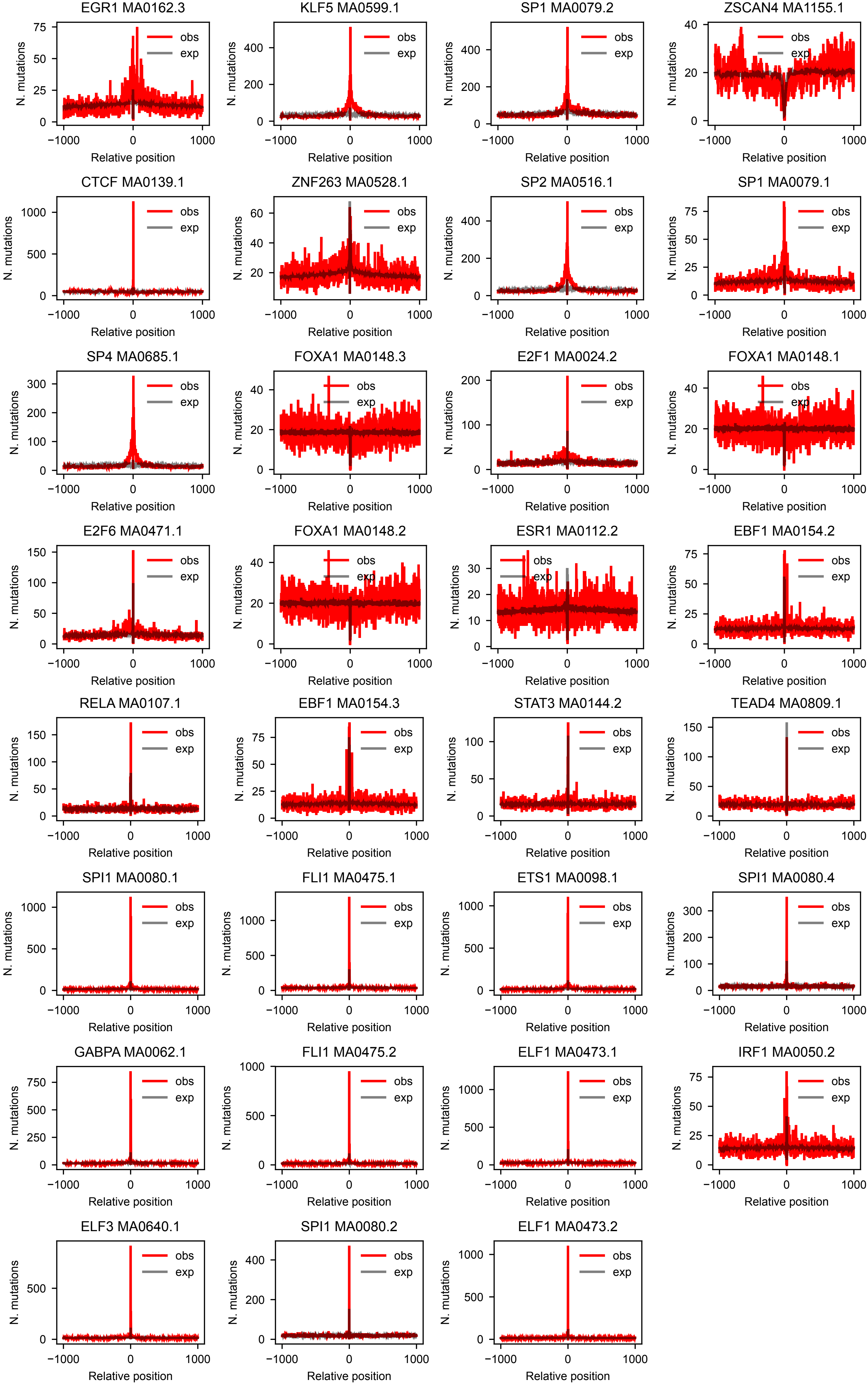
EGR1 MA0162.3
KLF5 MA0599.1
SP1 MA0079.2
ZSCAN4 MA1155.1
CTCF MA0139.1
ZNF263 MA0528.1
SP2 MA0516.1
SP1 MA0079.1
SP4 MA0685.1
FOXA1 MA0148.3
E2F1 MA0024.2
FOXA1 MA0148.1
E2F6 MA0471.1
FOXA1 MA0148.2
ESR1 MA0112.2
EBF1 MA0154.2
RELA MA0107.1
EBF1 MA0154.3
STAT3 MA0144.2
TEAD4 MA0809.1
SPI1 MA0080.1
FLI1 MA0475.1
ETS1 MA0098.1
SPI1 MA0080.4
GABPA MA0062.1
FLI1 MA0475.2
ELF1 MA0473.1
IRF1 MA0050.2
ELF3 MA0640.1
SPI1 MA0080.2
ELF1 MA0473.2
obs
exp
N. mutations
Relative position

Figure S4. Observed and expected mutation rates across the stacked 2001-nucleotide sequences across all transcription factors analyzed.
These graphs are equivalent to the examples shown in Figure 1a-d.

Figure S5

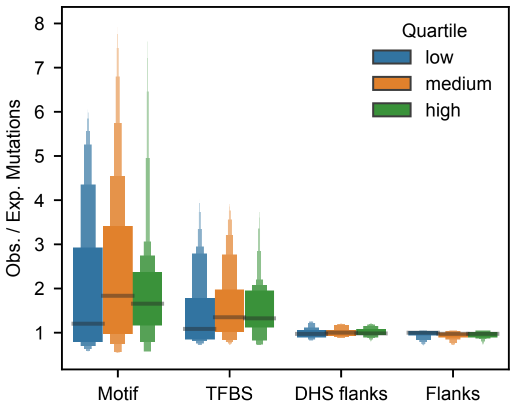
Quartile
low
medium
high
Obs. / Exp. Mutations
1
2
3
4
5
6
7
8
Motif
TFBS
DHS flanks
Flanks

Figure S5. Distribution of the ratio of observed to expected mutations (in log2 scale) across all TFBS grouped by tertiles of expression of their cognate TFs (within the TCGA skin cancer cohort).

The distributions for all regions of the TFBS (motif, TFBS, DHS flank and flanks) are shown separately.

Figure S6

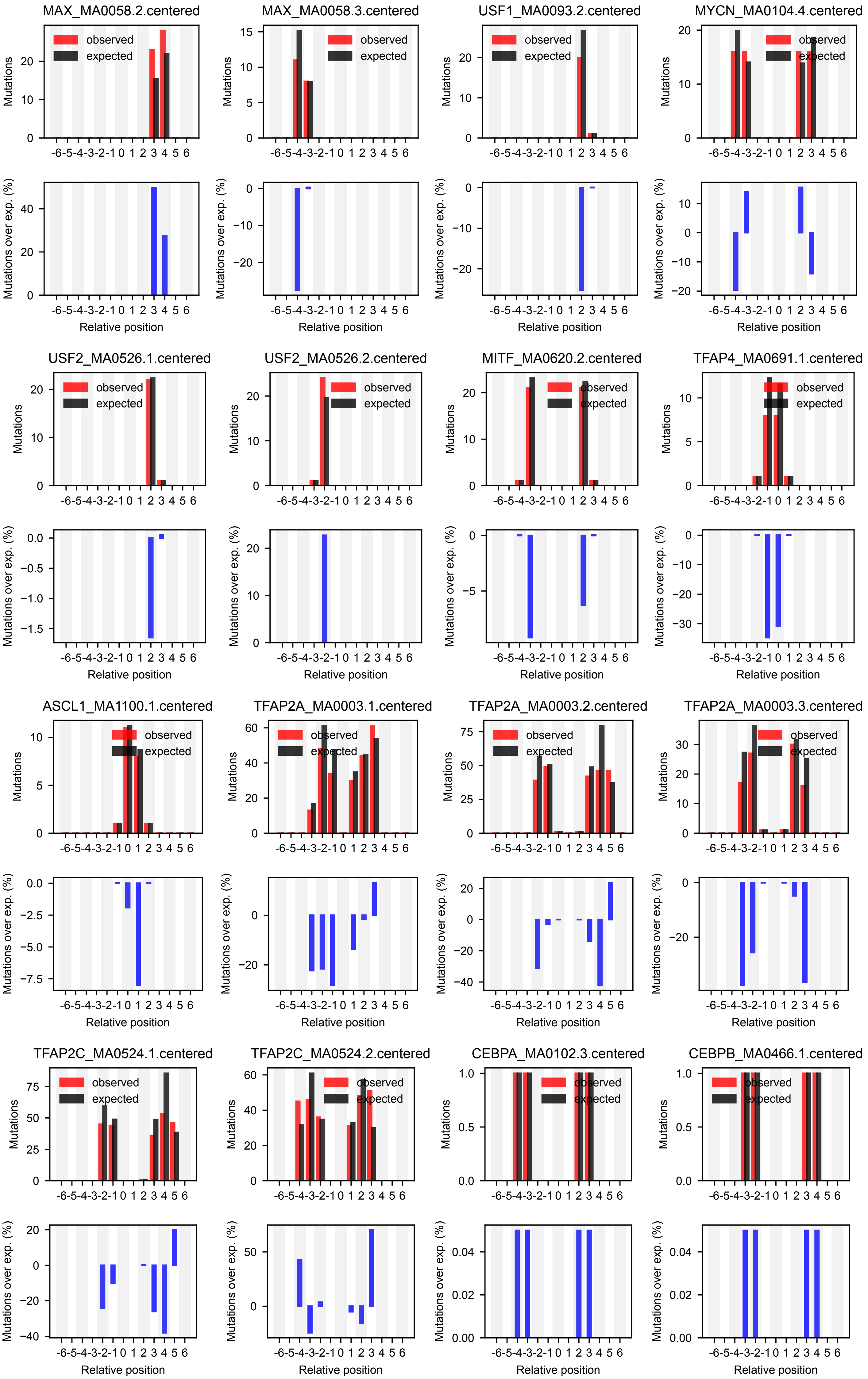
MAX_MA0058.2.centered
MAX_MA0058.3.centered
USF1_MA0093.2.centered
MYCN_MA0104.4.centered
USF2_MA0526.1.centered
USF2_MA0526.2.centered
MITF_MA0620.2.centered
TFAP4_MA0691.1.centered
ASCL1_MA1100.1.centered
TFAP2A_MA0003.1.centered
TFAP2A_MA0003.2.centered
TFAP2A_MA0003.3.centered
TFAP2C_MA0524.1.centered
TFAP2C_MA0524.2.centered
CEBPA_MA0102.3.centered
CEBPB_MA0466.1.centered
observed
expected
Mutations
Mutations over exp. (%)
-6-5-4-3-2-1 0 1 2 3 4 5 6
Relative position

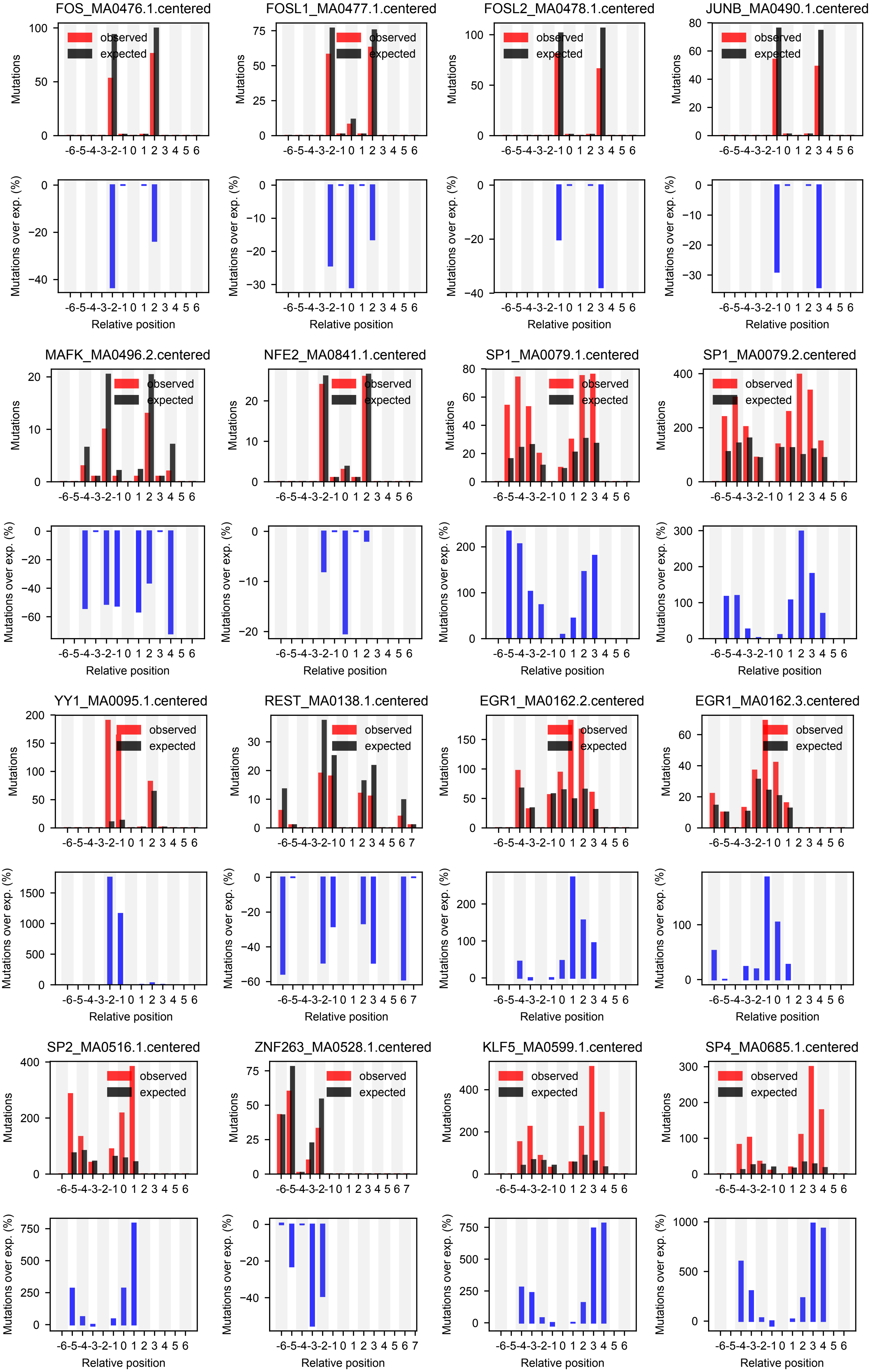
FOS_MA0476.1.centered
FOSL1_MA0477.1.centered
FOSL2_MA0478.1.centered
JUNB_MA0490.1.centered
MAFK_MA0496.2.centered
NFE2_MA0841.1.centered
SP1_MA0079.1.centered
SP1_MA0079.2.centered
YY1_MA0095.1.centered
REST_MA0138.1.centered
EGR1_MA0162.2.centered
EGR1_MA0162.3.centered
SP2_MA0516.1.centered
ZNF263_MA0528.1.centered
KLF5_MA0599.1.centered
SP4_MA0685.1.centered
observed
expected
Mutations
Mutations over exp. (%)
Relative position
-6-5-4-3-2-1 0 1 2 3 4 5 6

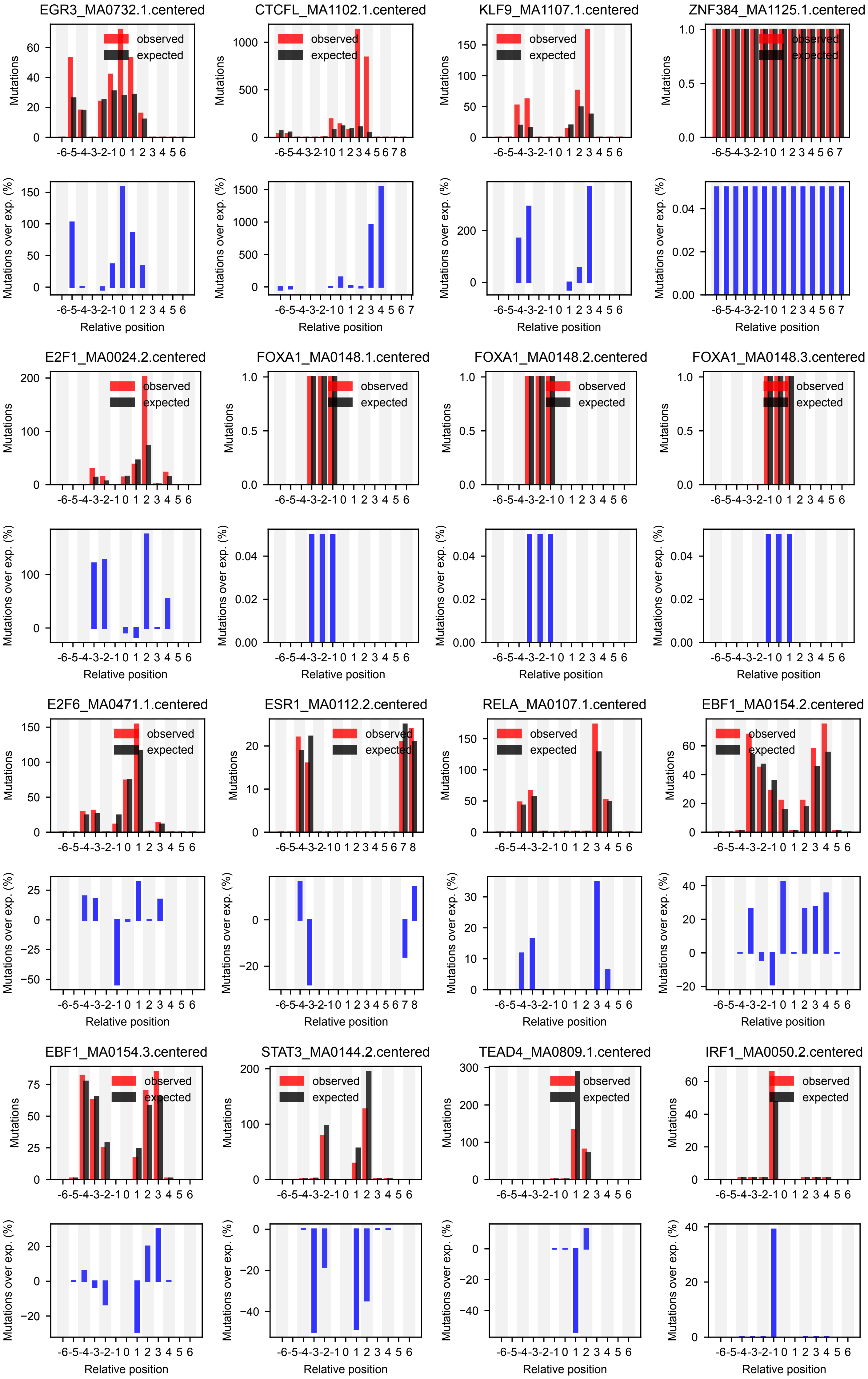
EGR3_MA0732.1.centered
CTCFL_MA1102.1.centered
KLF9_MA1107.1.centered
ZNF384_MA1125.1.centered
E2F1_MA0024.2.centered
FOXA1_MA0148.1.centered
FOXA1_MA0148.2.centered
FOXA1_MA0148.3.centered
E2F6_MA0471.1.centered
ESR1_MA0112.2.centered
RELA_MA0107.1.centered
EBF1_MA0154.2.centered
EBF1_MA0154.3.centered
STAT3_MA0144.2.centered
TEAD4_MA0809.1.centered
IRF1_MA0050.2.centered
observed
expected
Mutations
Mutations over exp. (%)
Relative position

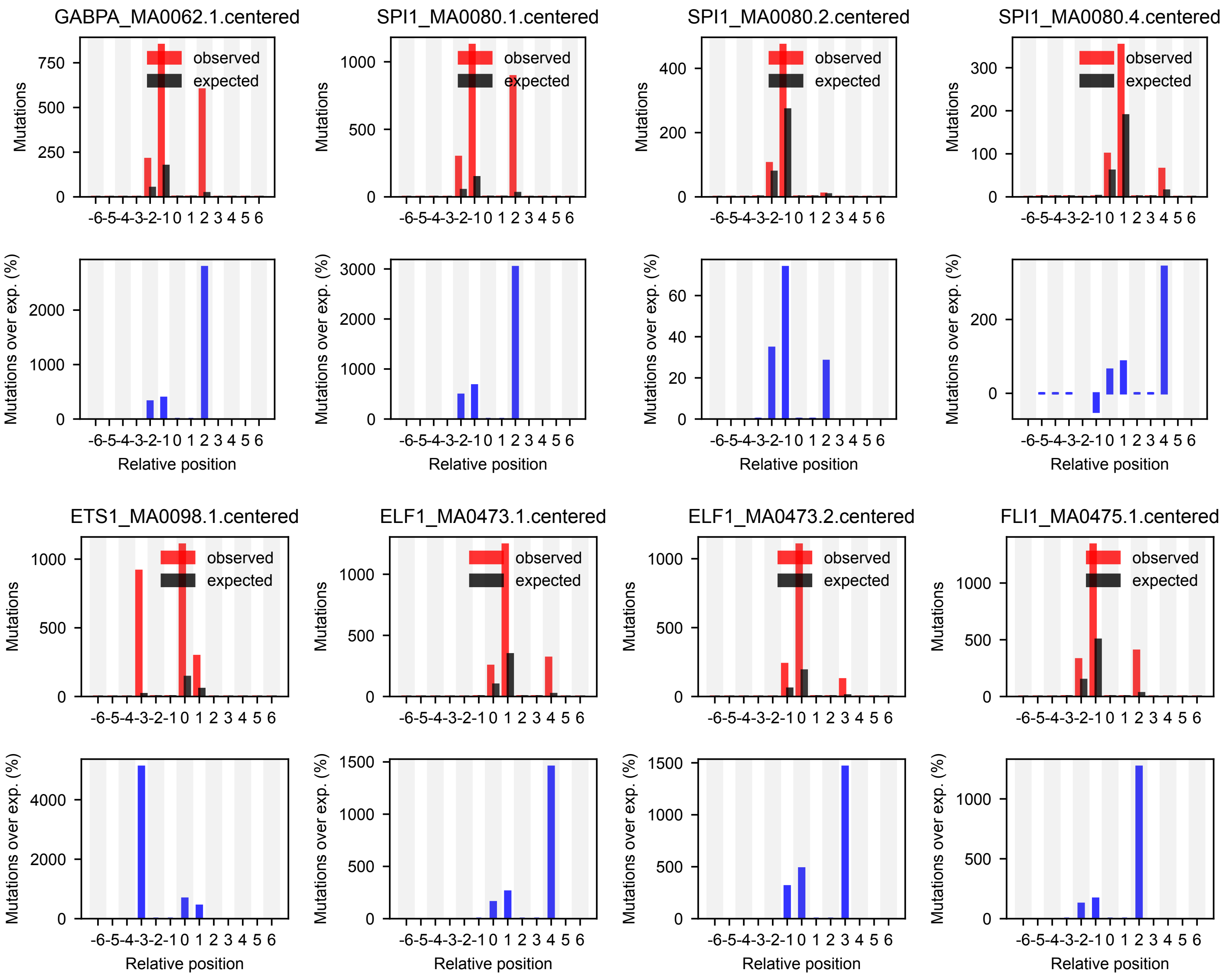
GABPA_MA0062.1.centered
SPI1_MA0080.1.centered
SPI1_MA0080.2.centered
SPI1_MA0080.4.centered
observed
expected
Mutations
Mutations over exp. (%)
Relative position
ETS1_MA0098.1.centered
ELF1_MA0473.1.centered
ELF1_MA0473.2.centered
FLI1_MA0475.1.centered

Figure S6. Observed (red) and expected (black) number of UV-induced mutations at dipyrimidines within all motifs, and percentage of increase or decrease with respect to the expectation (in blue).
These graphs are equivalent to the examples shown in Figure 2a-d.

Figure S7

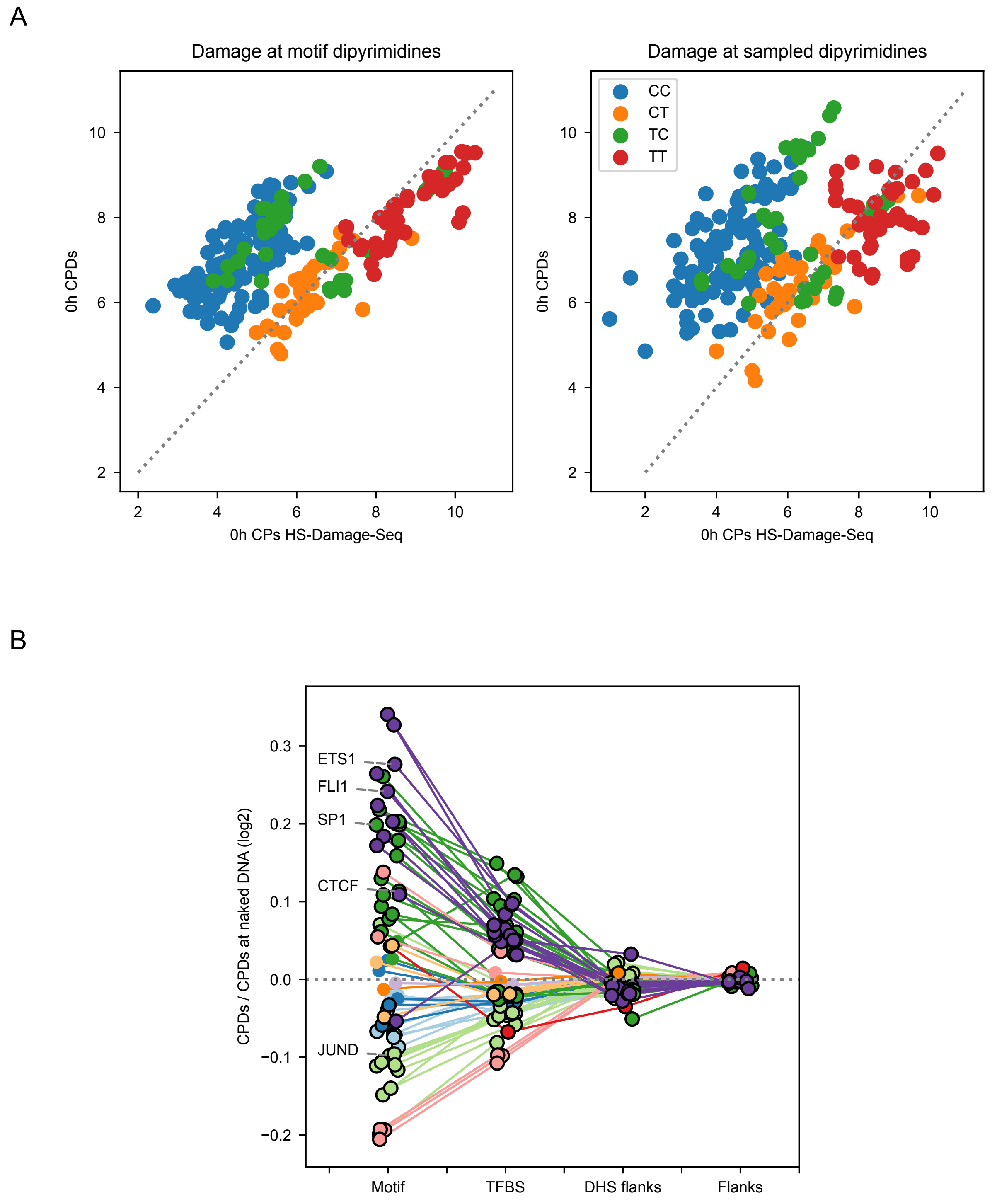

A
Damage at motif dipyrimidines
Damage at sampled dipyrimidines
CC
CT
TC
TT
0h CPDs
0h CPs HS-Damage-Seq
10
8
6
4
2
B
ETS1
FLI1
SP1
CTCF
JUND
CPDs / CPDs at naked DNA (log2)
0.3
0.2
0.1
0.0
−0.1
−0.2
Motif
TFBS
DHS flanks
Flanks

Figure S7. The robustness of results across different whole-genome CPD maps
(A) Several studies have mapped CPDs to the genome using different methodologies. We assessed the correlation between the number of CPDs of each type measured by Mao et al. (ref. 21) (y axis) and Hu et al. (ref. 30) (x axis), both within the binding motif (left) and at the flanks (right), and observed deviations from equivalency specific to CC and TC CPDs. This could be explained by differences in sequence specificity in the antibodies-based method.
(B) Ratio of observed to expected CPDs formation rates (in log2 scale) within the four regions studied (as in Fig. 3E), but using CPDs mapped by Elliot et al (ref. 22). Interestingly, the same trend of CPD formation rates within the TFBS are observed for several TF families (Tryptophan cluster and Basic leucine zipper) with respect to the analysis based on Mao et al (ref. 21) data shown in Figure 3E. However, the rate of CPD formation at the binding sites of TFs of other families appear different depending on the starting whole-genome CPD map employed. These differences may stem from differences in the experimental protocol (e.g., cells from different tissues, different irradiation protocol, and different sequencing platforms). All these differences may bias the starting CPD maps in different ways, which require further studies to clarify.

Figure S8

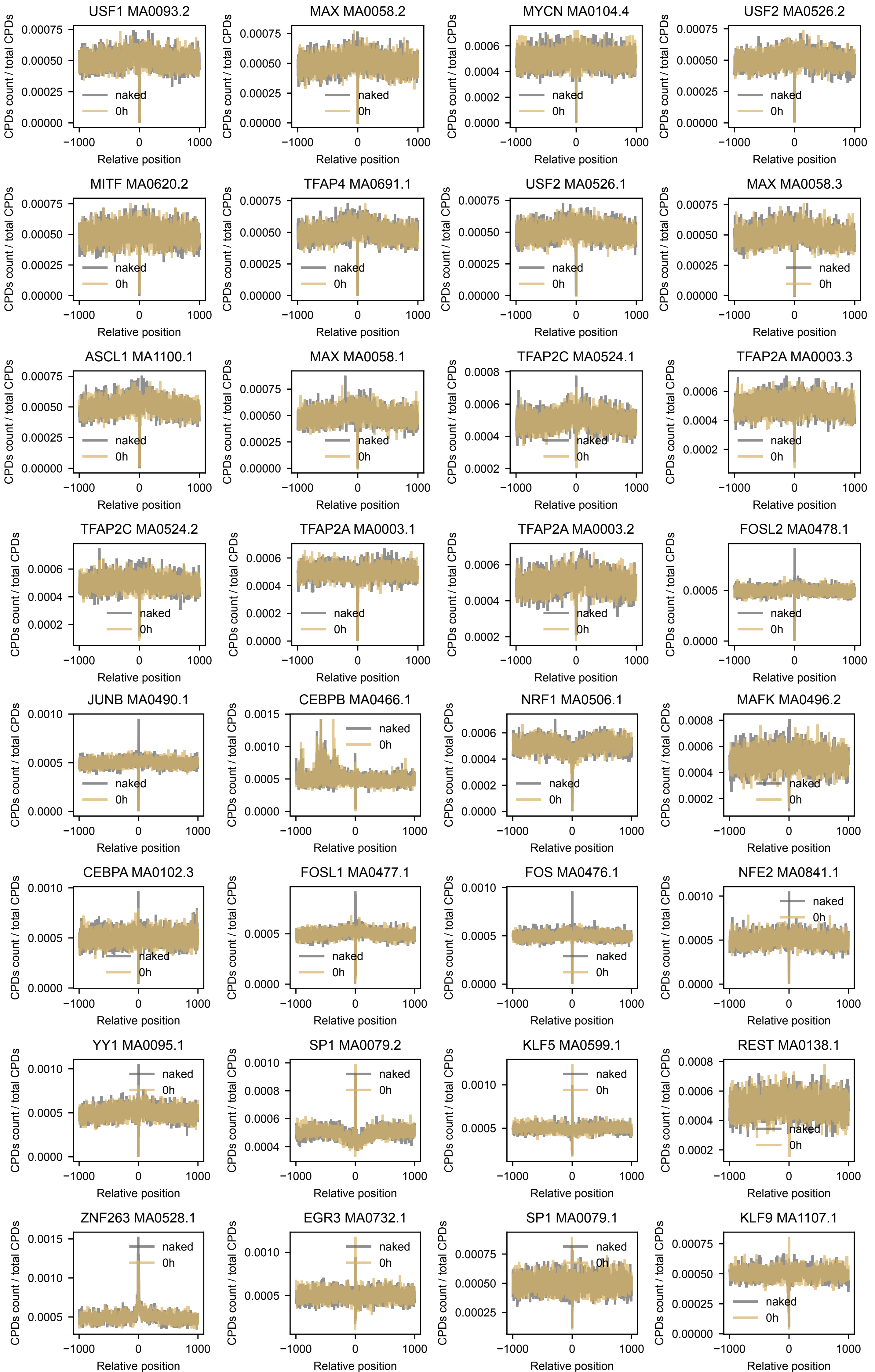
USF1 MA0093.2
MAX MA0058.2
MYCN MA0104.4
USF2 MA0526.2
MITF MA0620.2
TFAP4 MA0691.1
USF2 MA0526.1
MAX MA0058.3
ASCL1 MA1100.1
MAX MA0058.1
TFAP2C MA0524.1
TFAP2A MA0003.3
TFAP2C MA0524.2
TFAP2A MA0003.1
TFAP2A MA0003.2
FOSL2 MA0478.1
JUNB MA0490.1
CEBPB MA0466.1
NRF1 MA0506.1
MAFK MA0496.2
CEBPA MA0102.3
FOSL1 MA0477.1
FOS MA0476.1
NFE2 MA0841.1
YY1 MA0095.1
SP1 MA0079.2
KLF5 MA0599.1
REST MA0138.1
ZNF263 MA0528.1
EGR3 MA0732.1
SP1 MA0079.1
KLF9 MA1107.1
CPDs count / total CPDs
Relative position
naked
0h
−1000
0
1000

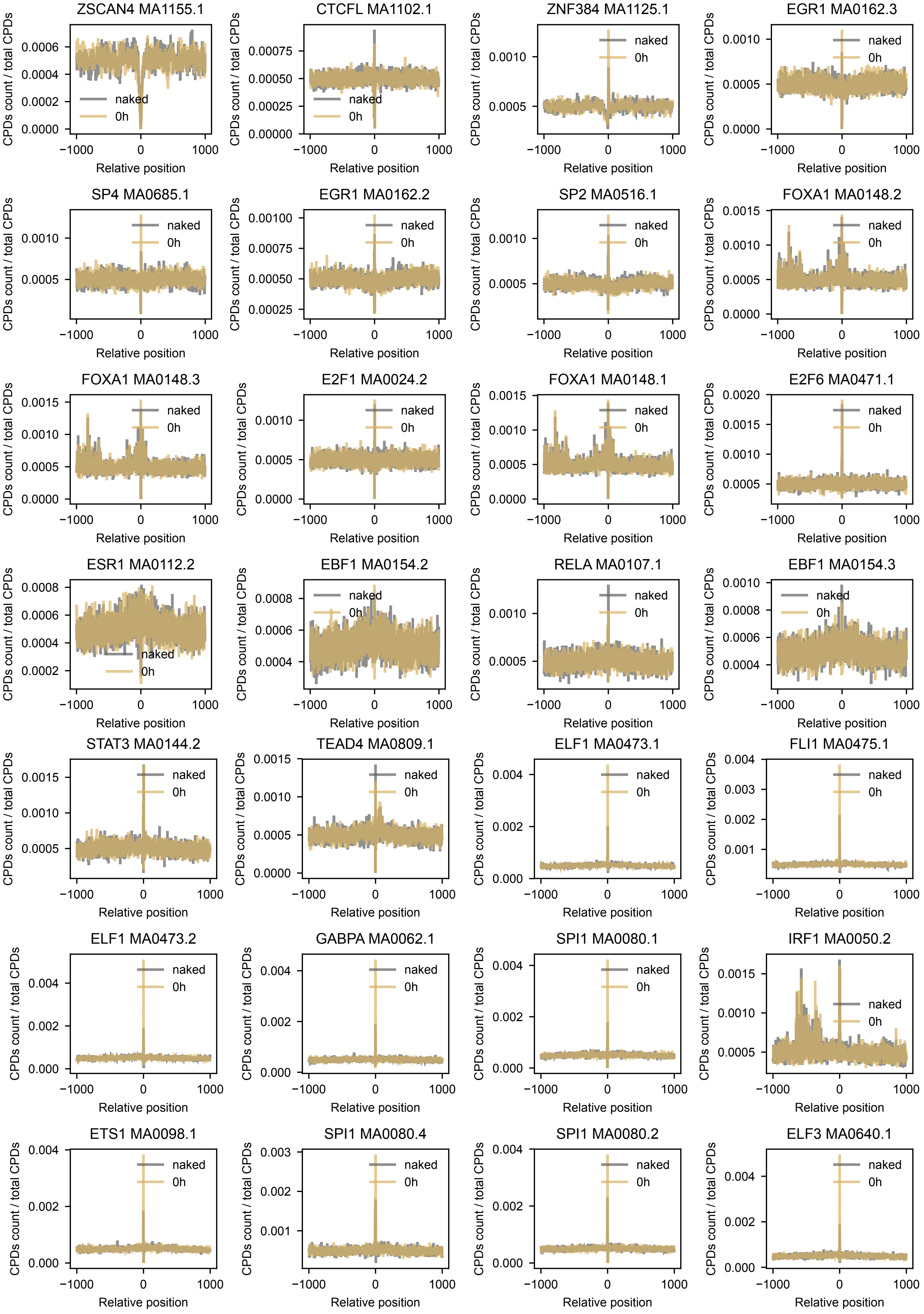
ZSCAN4 MA1155.1
CTCFL MA1102.1
ZNF384 MA1125.1
EGR1 MA0162.3
SP4 MA0685.1
EGR1 MA0162.2
SP2 MA0516.1
FOXA1 MA0148.2
FOXA1 MA0148.3
E2F1 MA0024.2
FOXA1 MA0148.1
E2F6 MA0471.1
ESR1 MA0112.2
EBF1 MA0154.2
RELA MA0107.1
EBF1 MA0154.3
STAT3 MA0144.2
TEAD4 MA0809.1
ELF1 MA0473.1
FLI1 MA0475.1
ELF1 MA0473.2
GABPA MA0062.1
SPI1 MA0080.1
IRF1 MA0050.2
ETS1 MA0098.1
SPI1 MA0080.4
SPI1 MA0080.2
ELF3 MA0640.1
CPDs count / total CPDs
Relative position
naked
0h

Figure S8. Distribution of the rate of formation of CPDs across the binding motifs of all TFs analyzed, with the TF bound to the DNA, and in naked DNA.
These graphs are equivalent to the examples shown in Figure 3a-d.

Figure S9

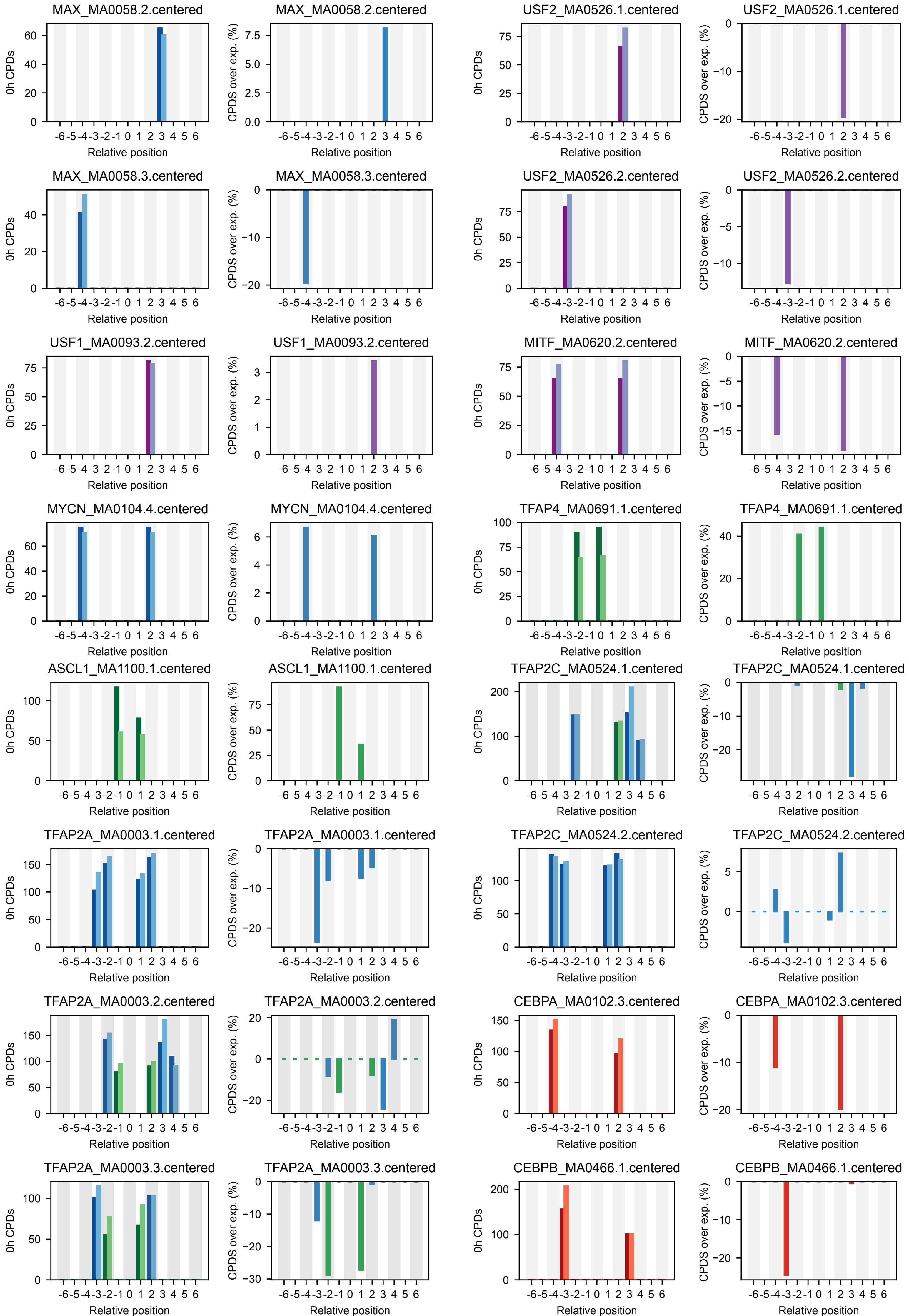
MAX_MA0058.2.centered
MAX_MA0058.2.centered
USF2_MA0526.1.centered
USF2_MA0526.1.centered
MAX_MA0058.3.centered
MAX_MA0058.3.centered
USF2_MA0526.2.centered
USF2_MA0526.2.centered
USF1_MA0093.2.centered
USF1_MA0093.2.centered
MITF_MA0620.2.centered
MITF_MA0620.2.centered
MYCN_MA0104.4.centered
MYCN_MA0104.4.centered
TFAP4_MA0691.1.centered
TFAP4_MA0691.1.centered
ASCL1_MA1100.1.centered
ASCL1_MA1100.1.centered
TFAP2C_MA0524.1.centered
TFAP2C_MA0524.1.centered
TFAP2A_MA0003.1.centered
TFAP2A_MA0003.1.centered
TFAP2C_MA0524.2.centered
TFAP2C_MA0524.2.centered
TFAP2A_MA0003.2.centered
TFAP2A_MA0003.2.centered
CEBPA_MA0102.3.centered
CEBPA_MA0102.3.centered
TFAP2A_MA0003.3.centered
TFAP2A_MA0003.3.centered
CEBPB_MA0466.1.centered
CEBPB_MA0466.1.centered
0h CPDs
CPDS over exp. (%)
Relative position

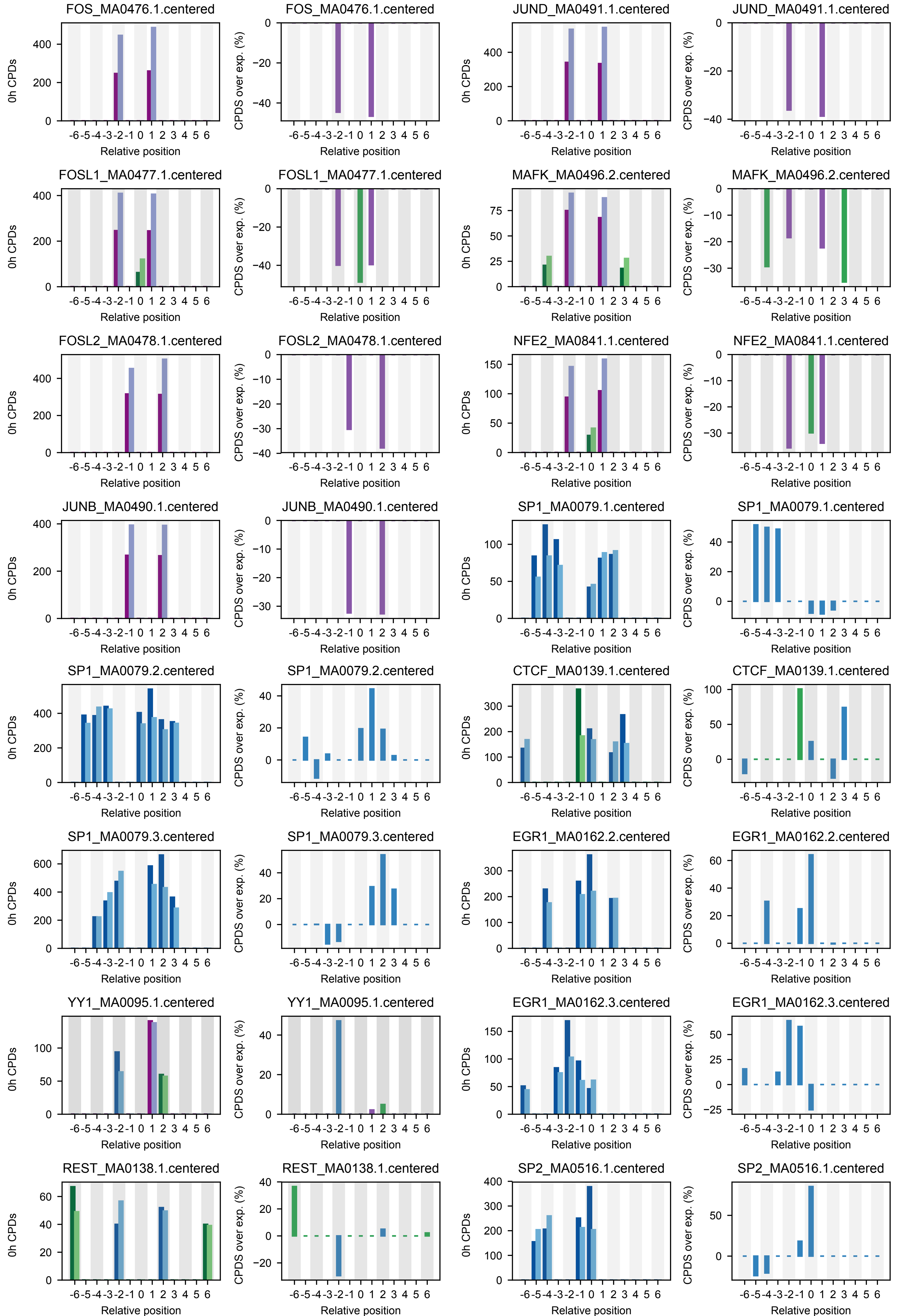
FOS_MA0476.1.centered
0h CPDs
CPDS over exp. (%)
JUND_MA0491.1.centered
FOSL1_MA0477.1.centered
MAFK_MA0496.2.centered
FOSL2_MA0478.1.centered
NFE2_MA0841.1.centered
JUNB_MA0490.1.centered
SP1_MA0079.1.centered
SP1_MA0079.2.centered
CTCF_MA0139.1.centered
SP1_MA0079.3.centered
EGR1_MA0162.2.centered
YY1_MA0095.1.centered
EGR1_MA0162.3.centered
REST_MA0138.1.centered
SP2_MA0516.1.centered
Relative position
-6 -5 -4 -3 -2 -1 0 1 2 3 4 5 6

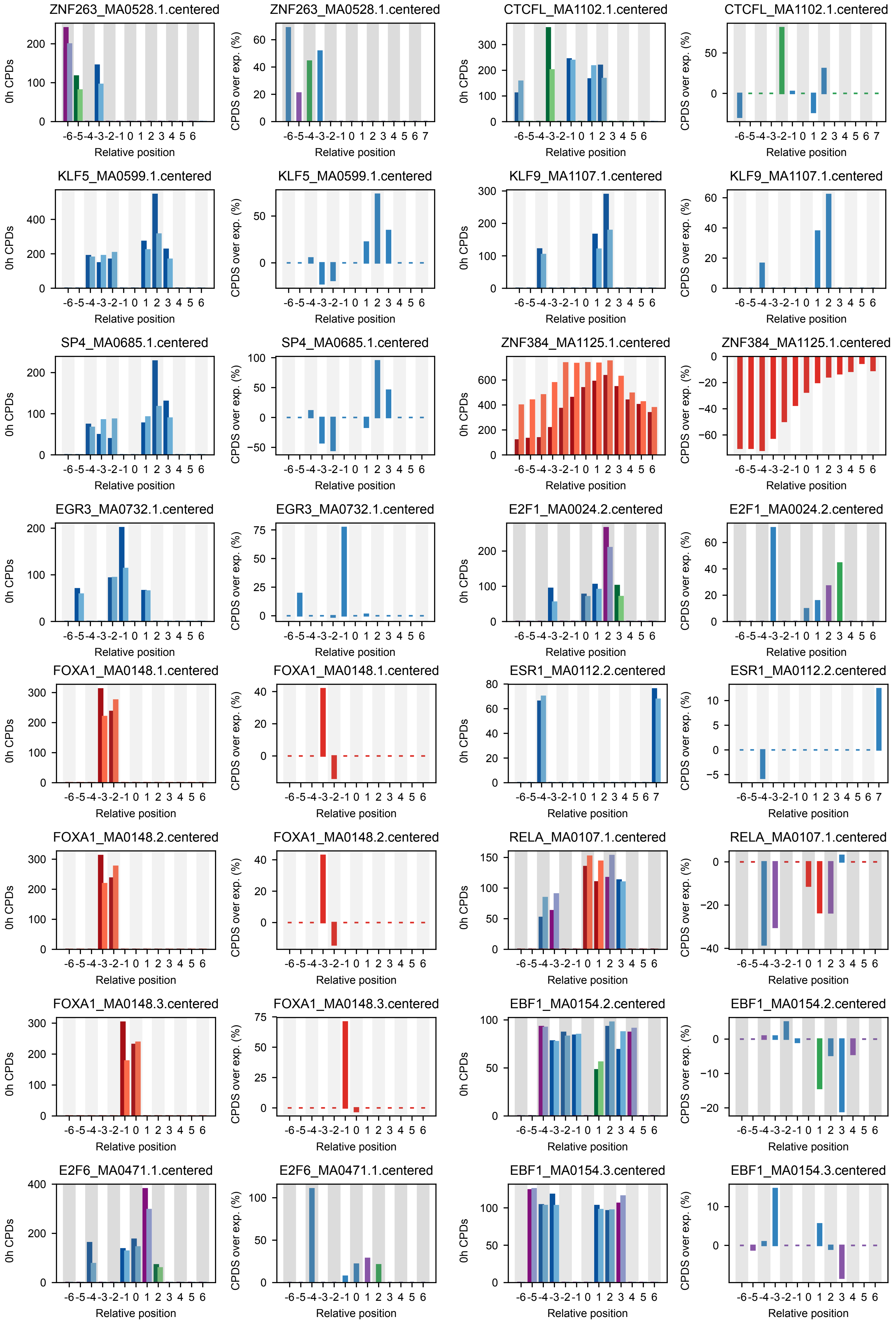
ZNF263_MA0528.1.centered
CTCFL_MA1102.1.centered
KLF5_MA0599.1.centered
KLF9_MA1107.1.centered
SP4_MA0685.1.centered
ZNF384_MA1125.1.centered
EGR3_MA0732.1.centered
E2F1_MA0024.2.centered
FOXA1_MA0148.1.centered
ESR1_MA0112.2.centered
FOXA1_MA0148.2.centered
RELA_MA0107.1.centered
FOXA1_MA0148.3.centered
EBF1_MA0154.2.centered
E2F6_MA0471.1.centered
EBF1_MA0154.3.centered
0h CPDs
CPDS over exp. (%)
Relative position

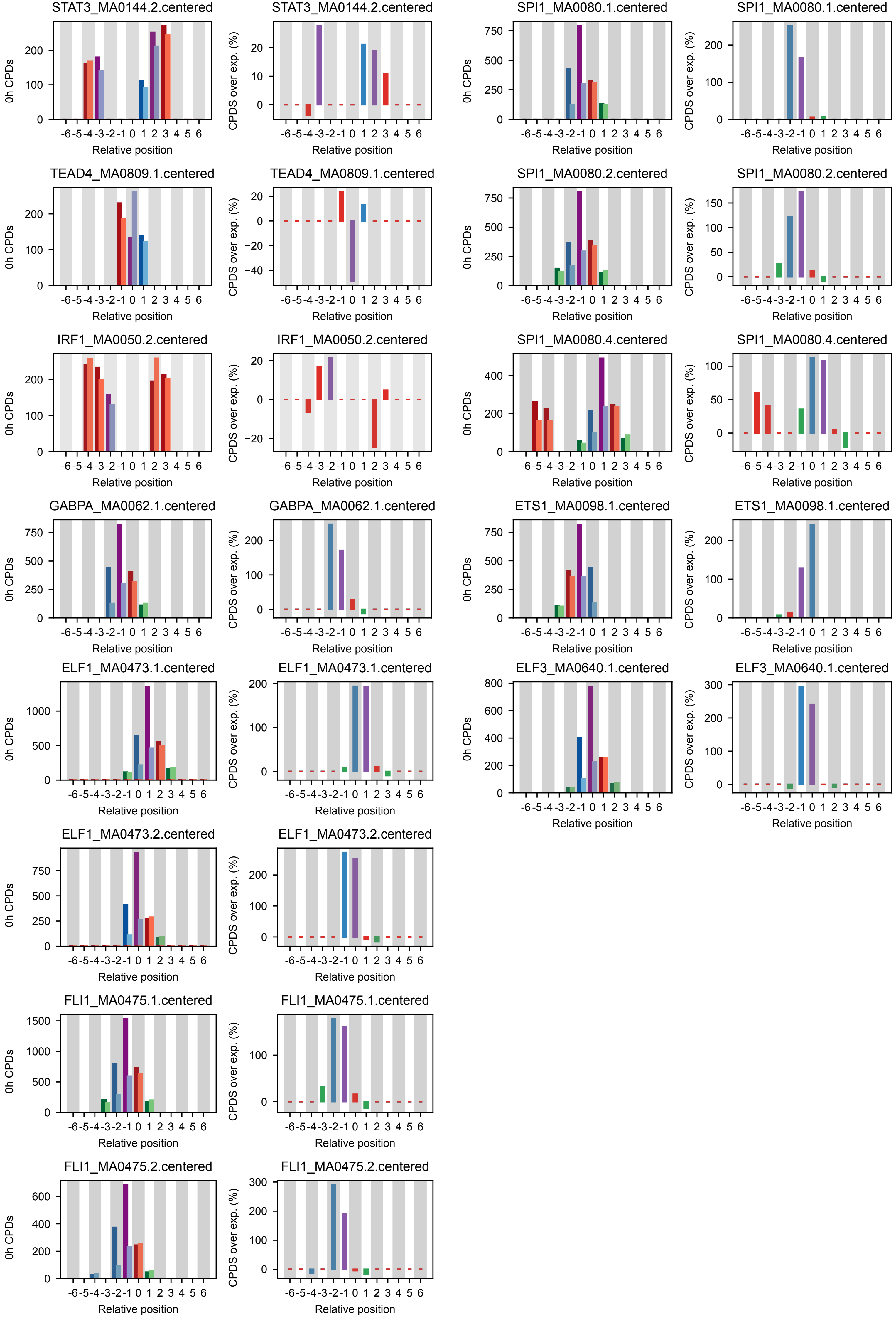

STAT3_MA0144.2.centered
0h CPDs
CPDS over exp. (%)
SPI1_MA0080.1.centered
TEAD4_MA0809.1.centered
SPI1_MA0080.2.centered
IRF1_MA0050.2.centered
SPI1_MA0080.4.centered
GABPA_MA0062.1.centered
ETS1_MA0098.1.centered
ELF1_MA0473.1.centered
ELF3_MA0640.1.centered
ELF1_MA0473.2.centered
FLI1_MA0475.1.centered
FLI1_MA0475.2.centered
-6 -5 -4 -3 -2 -1 0 1 2 3 4 5 6
Relative position

Figure S9. For each transcription factor, the first panel shows CPDs formed at each dipyrimidine position within the binding motifs (dark) and in the flanks (light), which are formed by positions with identical sequence context.
The second panel shows the percentage of CPDs increase or decrease with respect to this expectation.

Figure S10

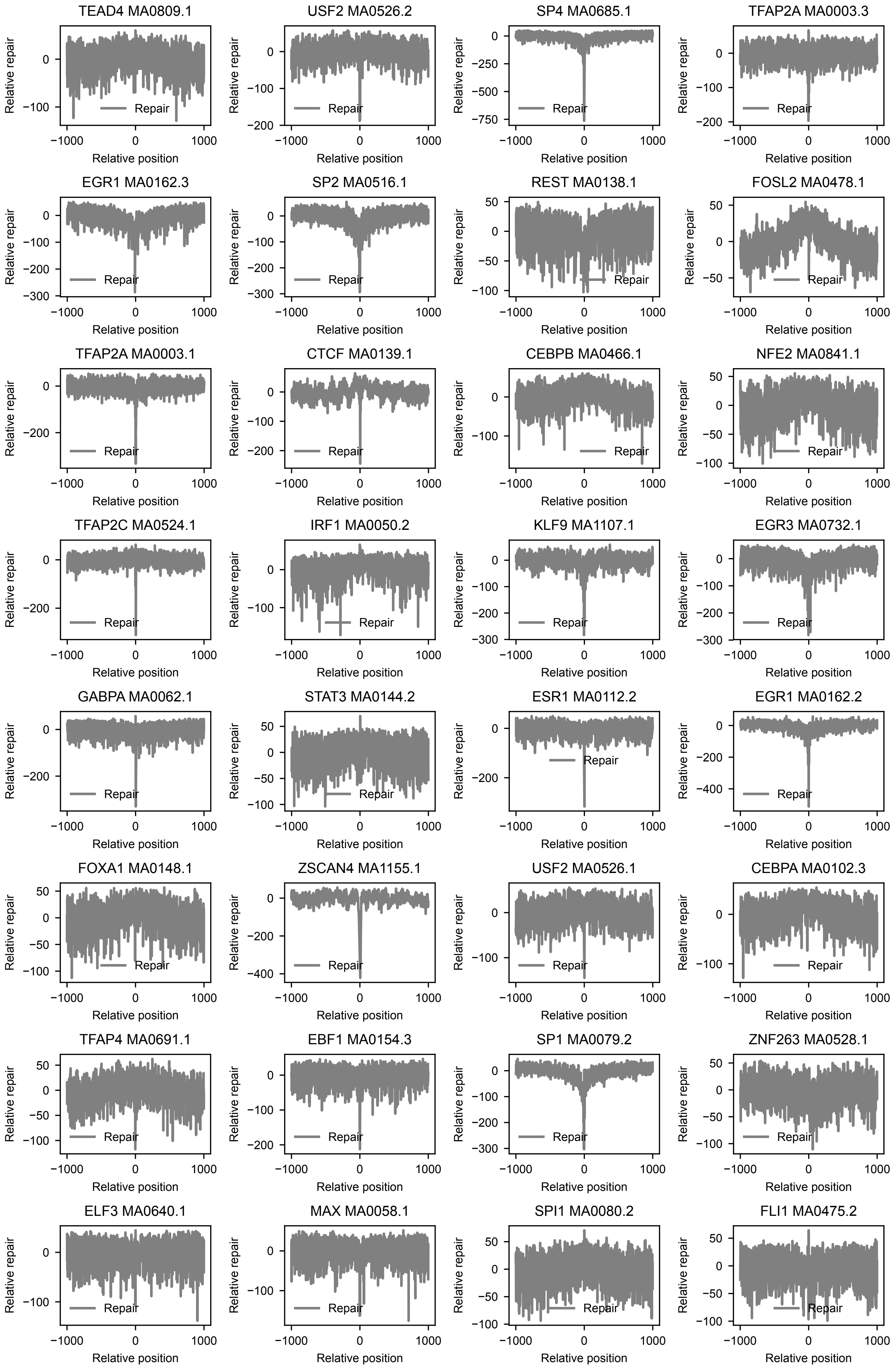

TEAD4 MA0809.1
USF2 MA0526.2
SP4 MA0685.1
TFAP2A MA0003.3
EGR1 MA0162.3
SP2 MA0516.1
REST MA0138.1
FOSL2 MA0478.1
TFAP2A MA0003.1
CTCF MA0139.1
CEBPB MA0466.1
NFE2 MA0841.1
TFAP2C MA0524.1
IRF1 MA0050.2
KLF9 MA1107.1
EGR3 MA0732.1
GABPA MA0062.1
STAT3 MA0144.2
ESR1 MA0112.2
EGR1 MA0162.2
FOXA1 MA0148.1
ZSCAN4 MA1155.1
USF2 MA0526.1
CEBPA MA0102.3
TFAP4 MA0691.1
EBF1 MA0154.3
SP1 MA0079.2
ZNF263 MA0528.1
ELF3 MA0640.1
MAX MA0058.1
SPI1 MA0080.2
FLI1 MA0475.2
Relative repair
Relative position
Repair

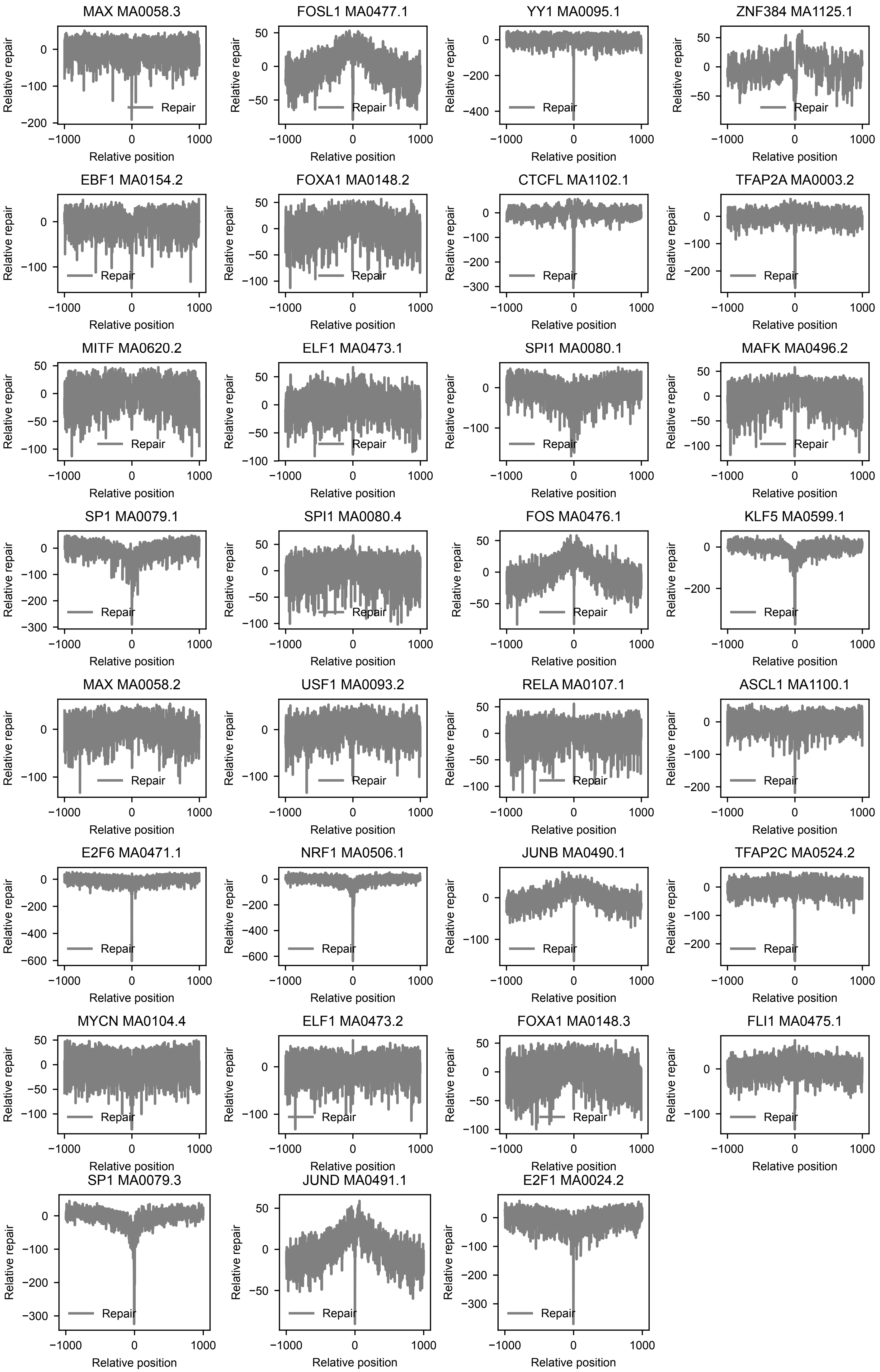
MAX MA0058.3
FOSL1 MA0477.1
YY1 MA0095.1
ZNF384 MA1125.1
EBF1 MA0154.2
FOXA1 MA0148.2
CTCFL MA1102.1
TFAP2A MA0003.2
MITF MA0620.2
ELF1 MA0473.1
SPI1 MA0080.1
MAFK MA0496.2
SP1 MA0079.1
SPI1 MA0080.4
FOS MA0476.1
KLF5 MA0599.1
MAX MA0058.2
USF1 MA0093.2
RELA MA0107.1
ASCL1 MA1100.1
E2F6 MA0471.1
NRF1 MA0506.1
JUNB MA0490.1
TFAP2C MA0524.2
MYCN MA0104.4
ELF1 MA0473.2
FOXA1 MA0148.3
FLI1 MA0475.1
SP1 MA0079.3
JUND MA0491.1
E2F1 MA0024.2
Relative repair
Relative position
Repair

Figure S10. CPDs repaired 48h after UV exposure across the stacked 2001-nucleotide sequences across for all transcription factors analyzed.
These graphs are equivalent to the second plot of the examples shown in Figure 4a-d.

Figure S11

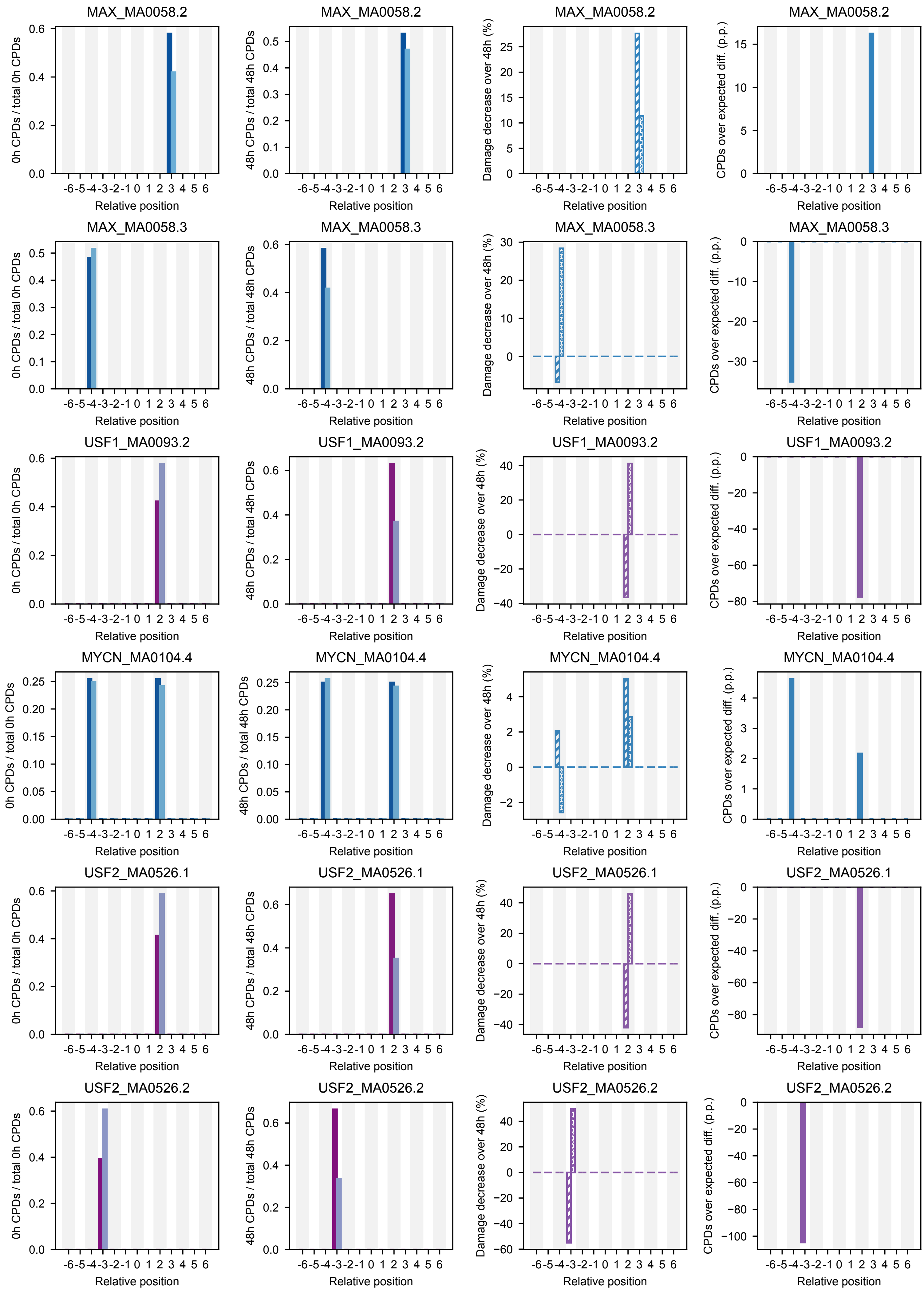
MAX_MA0058.2
MAX_MA0058.3
USF1_MA0093.2
MYCN_MA0104.4
USF2_MA0526.1
USF2_MA0526.2
0h CPDs / total 0h CPDs
48h CPDs / total 48h CPDs
Damage decrease over 48h (%)
CPDs over expected diff. (p.p.)
Relative position
-6 -5 -4 -3 -2 -1 0 1 2 3 4 5 6

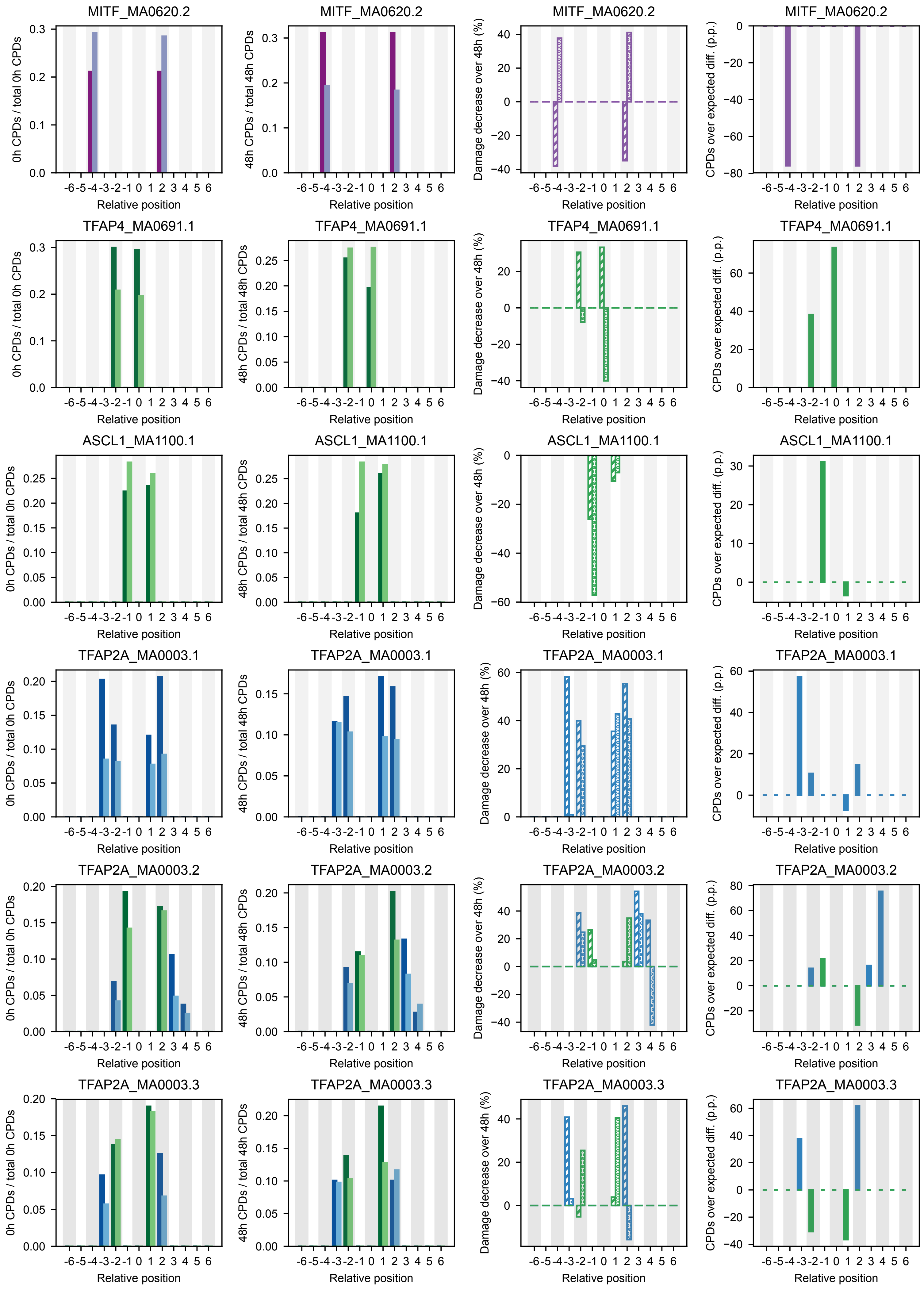
MITF_MA0620.2
0h CPDs / total 0h CPDs
48h CPDs / total 48h CPDs
Damage decrease over 48h (%)
CPDs over expected diff. (p.p.)
TFAP4_MA0691.1
ASCL1_MA1100.1
TFAP2A_MA0003.1
TFAP2A_MA0003.2
TFAP2A_MA0003.3
-6 -5 -4 -3 -2 -1 0 1 2 3 4 5 6
Relative position

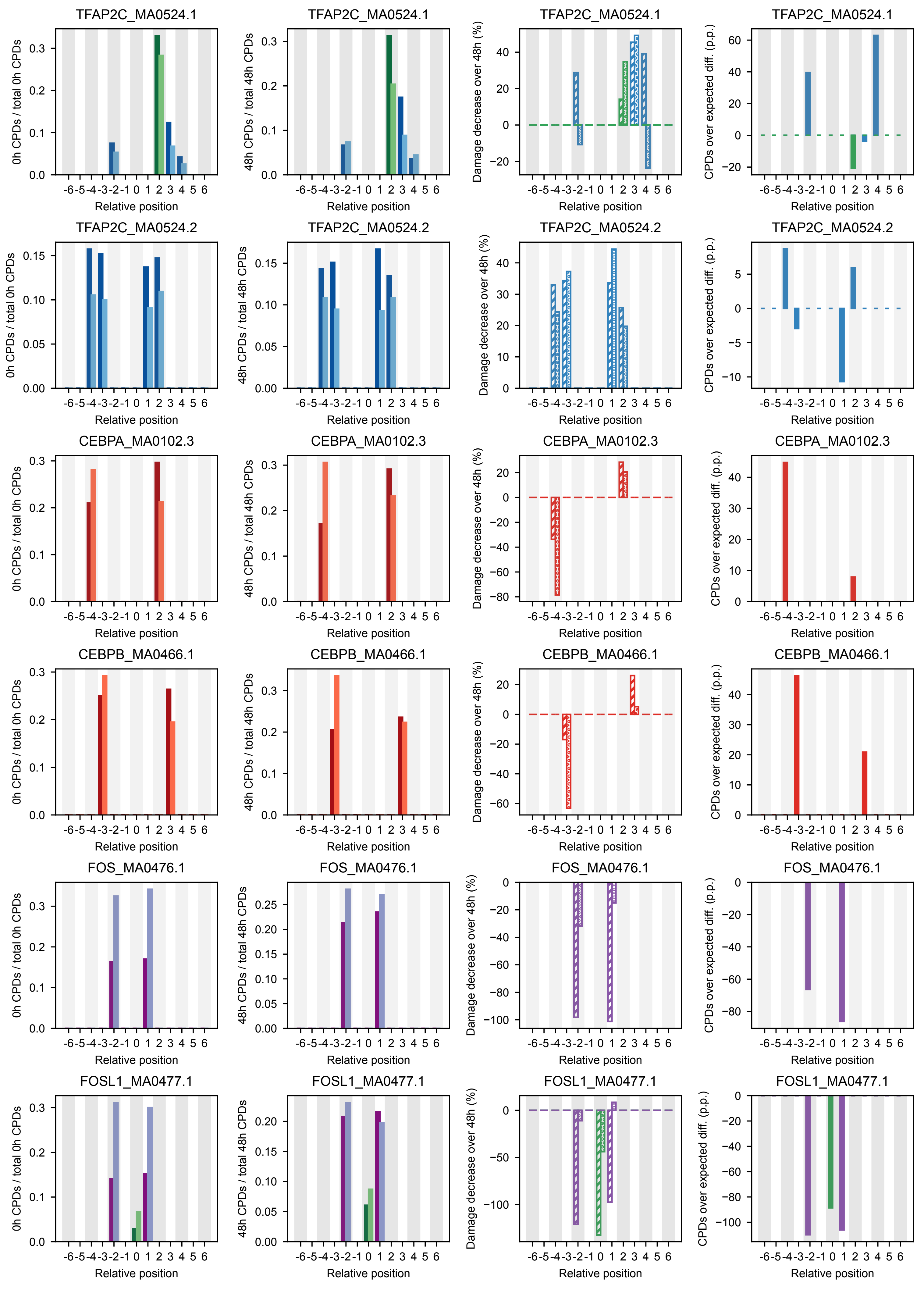

TFAP2C_MA0524.1
TFAP2C_MA0524.2
CEBPA_MA0102.3
CEBPB_MA0466.1
FOS_MA0476.1
FOSL1_MA0477.1
0h CPDs / total 0h CPDs
48h CPDs / total 48h CPDs
Damage decrease over 48h (%)
CPDs over expected diff. (p.p.)
Relative position
-6 -5 -4 -3 -2 -1 0 1 2 3 4 5 6

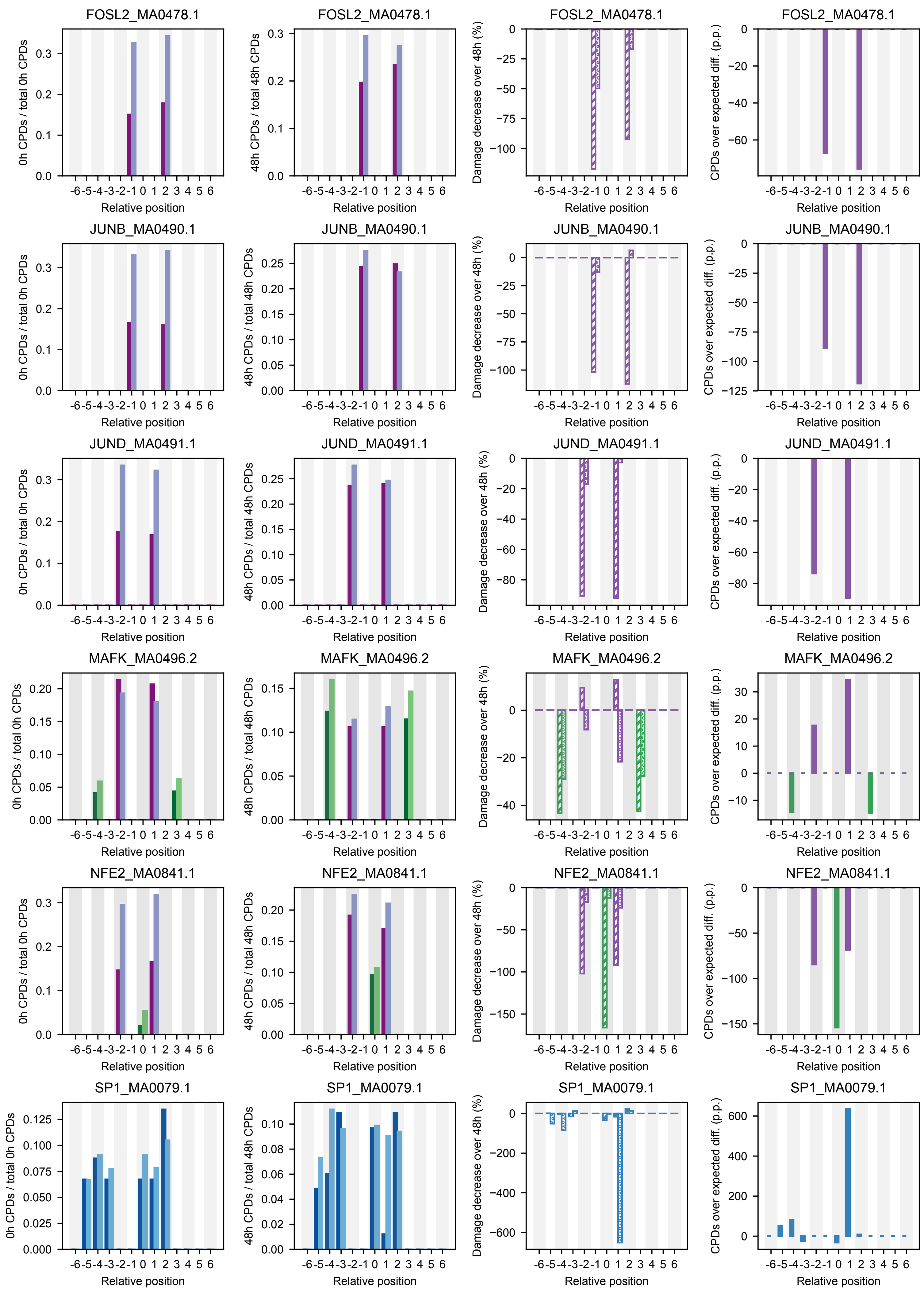
FOSL2_MA0478.1
0h CPDs / total 0h CPDs
48h CPDs / total 48h CPDs
Damage decrease over 48h (%)
CPDs over expected diff. (p.p.)
Relative position
-6-5-4-3-2-1 0 1 2 3 4 5 6
JUNB_MA0490.1
JUND_MA0491.1
MAFK_MA0496.2
NFE2_MA0841.1
SP1_MA0079.1

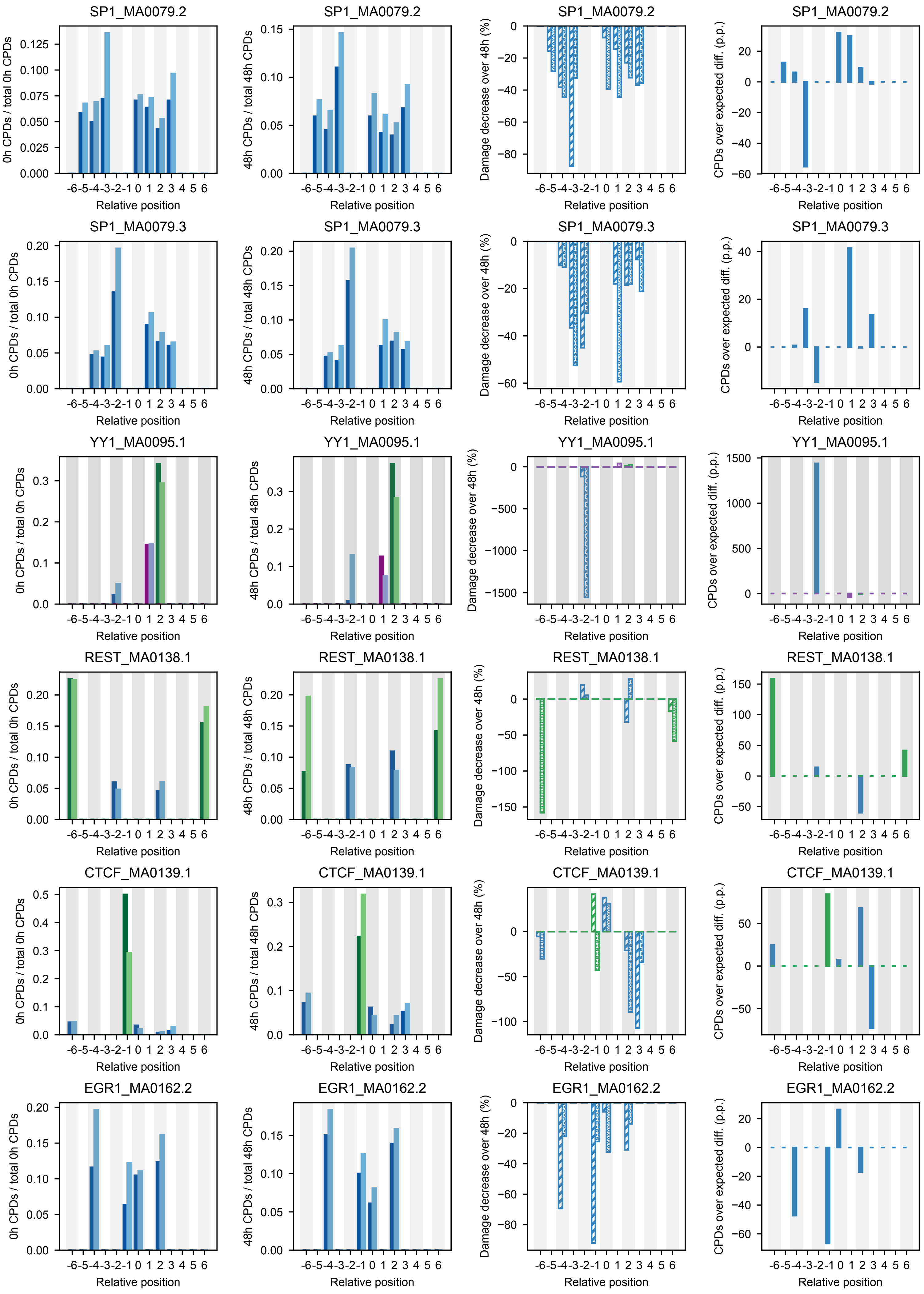
SP1_MA0079.2
0h CPDs / total 0h CPDs
48h CPDs / total 48h CPDs
Damage decrease over 48h (%)
CPDs over expected diff. (p.p.)
SP1_MA0079.3
YY1_MA0095.1
REST_MA0138.1
CTCF_MA0139.1
EGR1_MA0162.2
-6-5-4-3-2-1 0 1 2 3 4 5 6
Relative position

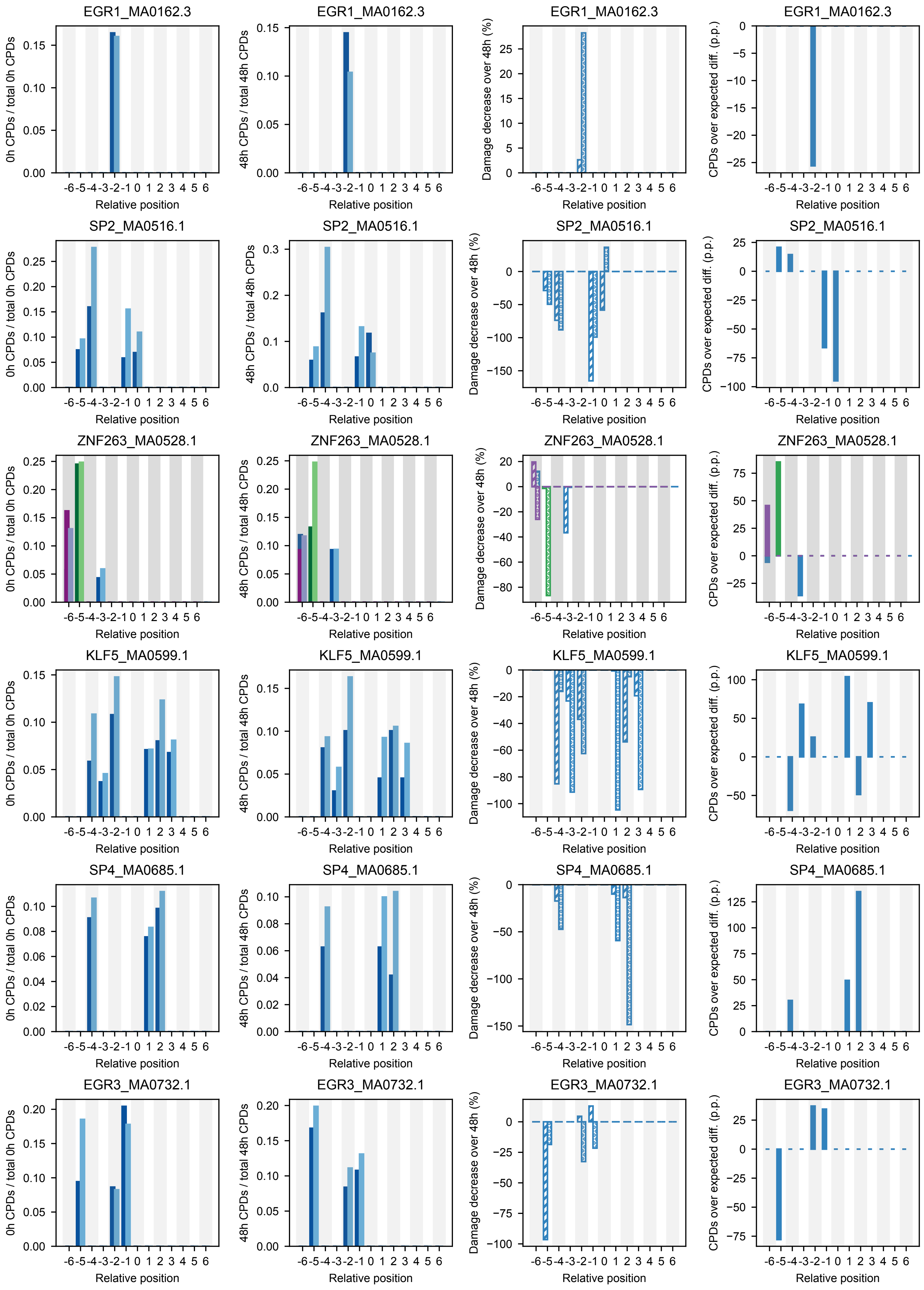
EGR1_MA0162.3
0h CPDs / total 0h CPDs
48h CPDs / total 48h CPDs
Damage decrease over 48h (%)
CPDs over expected diff. (p.p.)
SP2_MA0516.1
ZNF263_MA0528.1
KLF5_MA0599.1
SP4_MA0685.1
EGR3_MA0732.1
-6 -5 -4 -3 -2 -1 0 1 2 3 4 5 6
Relative position

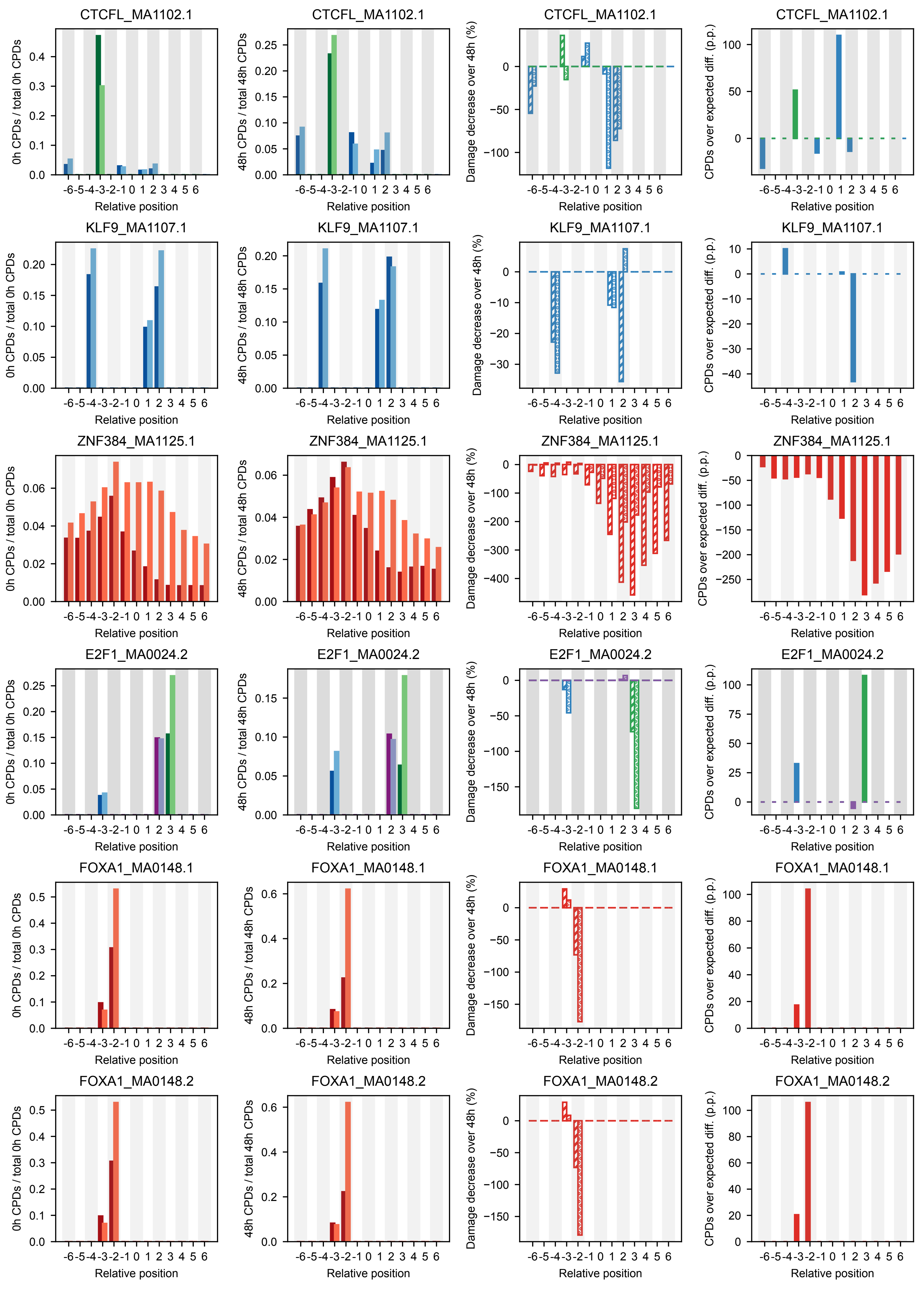
CTCFL_MA1102.1
0h CPDs / total 0h CPDs
48h CPDs / total 48h CPDs
Damage decrease over 48h (%)
CPDs over expected diff. (p.p.)
KLF9_MA1107.1
ZNF384_MA1125.1
E2F1_MA0024.2
FOXA1_MA0148.1
FOXA1_MA0148.2
-6-5-4-3-2-1 0 1 2 3 4 5 6
Relative position

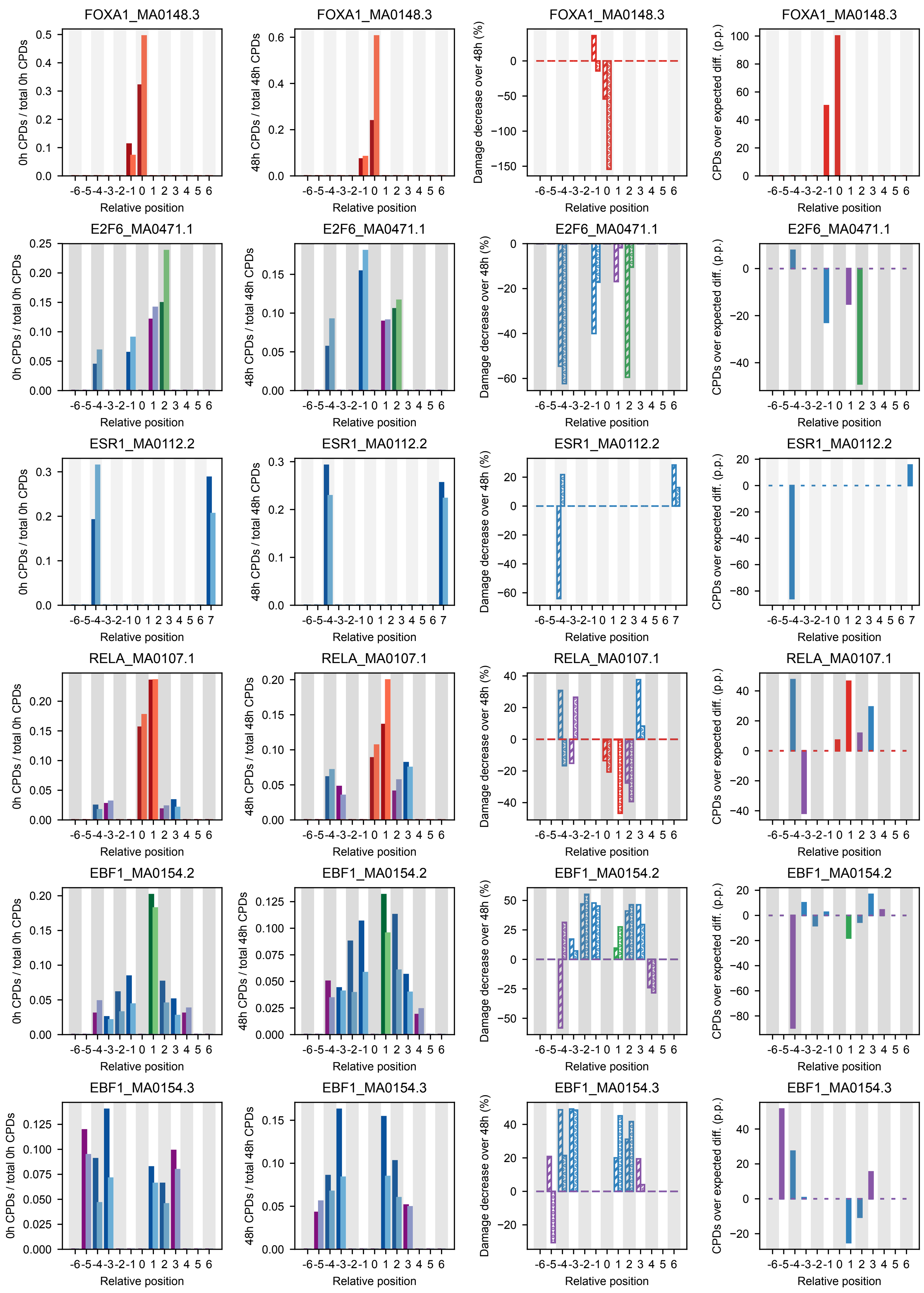
FOXA1_MA0148.3
0h CPDs / total 0h CPDs
48h CPDs / total 48h CPDs
Damage decrease over 48h (%)
CPDs over expected diff. (p.p.)
E2F6_MA0471.1
ESR1_MA0112.2
RELA_MA0107.1
EBF1_MA0154.2
EBF1_MA0154.3
Relative position

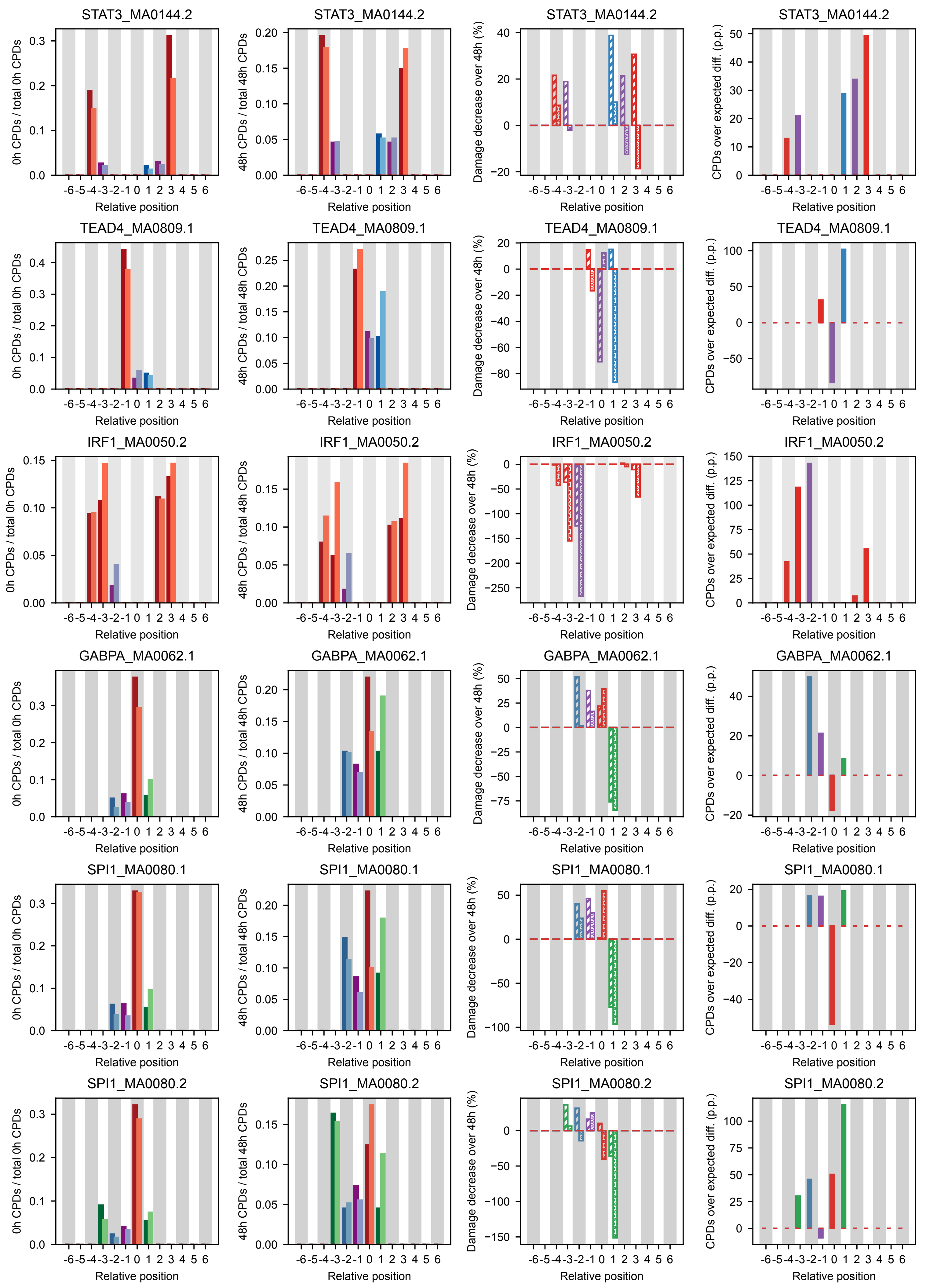
STAT3_MA0144.2
TEAD4_MA0809.1
IRF1_MA0050.2
GABPA_MA0062.1
SPI1_MA0080.1
SPI1_MA0080.2
0h CPDs / total 0h CPDs
48h CPDs / total 48h CPDs
Damage decrease over 48h (%)
CPDs over expected diff. (p.p.)
Relative position

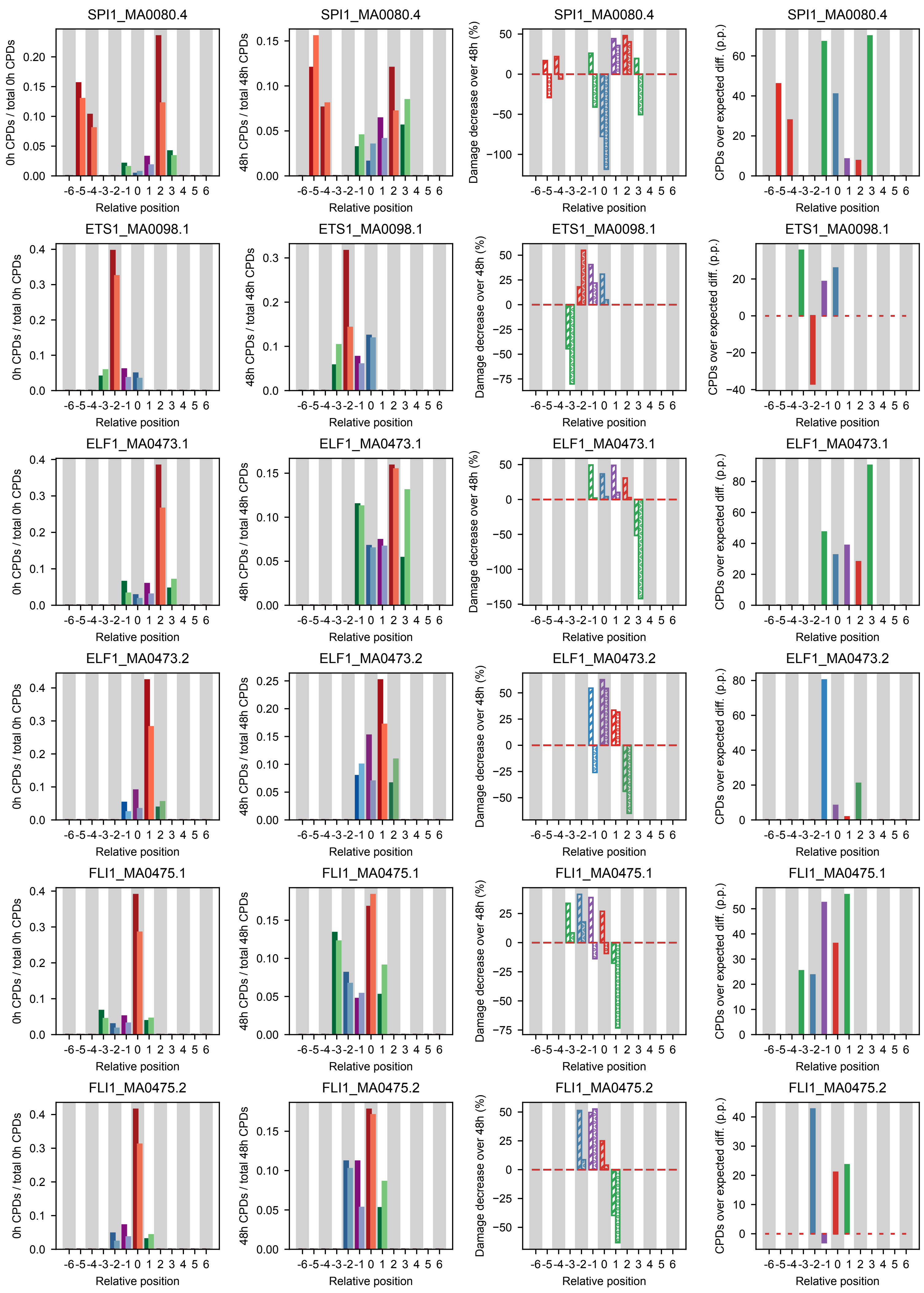
SPI1_MA0080.4
0h CPDs / total 0h CPDs
48h CPDs / total 48h CPDs
Damage decrease over 48h (%)
CPDs over expected diff. (p.p.)
ETS1_MA0098.1
ELF1_MA0473.1
ELF1_MA0473.2
FLI1_MA0475.1
FLI1_MA0475.2
-6 -5 -4 -3 -2 -1 0 1 2 3 4 5 6
Relative position

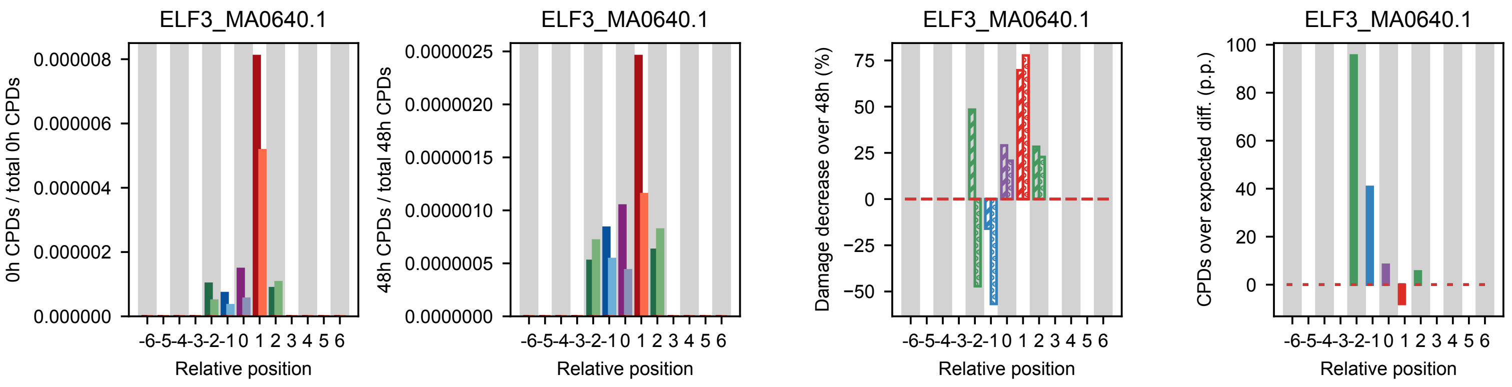
ELF3_MA0640.1
0h CPDs / total 0h CPDs
0.000008
0.000006
0.000004
0.000002
0.000000
-6-5-4-3-2-1 0 1 2 3 4 5 6
Relative position
ELF3_MA0640.1
48h CPDs / total 48h CPDs
0.0000025
0.0000020
0.0000015
0.0000010
0.0000005
0.0000000
-6-5-4-3-2-1 0 1 2 3 4 5 6
Relative position
ELF3_MA0640.1
Damage decrease over 48h (%)
75
50
25
0
−25
−50
-6-5-4-3-2-1 0 1 2 3 4 5 6
Relative position
ELF3_MA0640.1
CPDs over expected diff. (p.p.)
100
80
60
40
20
0
-6-5-4-3-2-1 0 1 2 3 4 5 6
Relative position

Figure S11. From left to right: 0h CPDs at motif dipyrimidines and at flanks (with equal sequence context).
CPDs count per position has been divided by the total number of CPDs at 0h. 48h CPDs at motif dipyrimidines and at flanks (with equal sequence context). CPDs count per position has been divided by the total number of CPDs at 48h. Relative repair at each position within the binding motif and in the flanks after 48h. Difference (in percentage points) between the relative repair in the motif and in the flanks, per position.

Figure S12

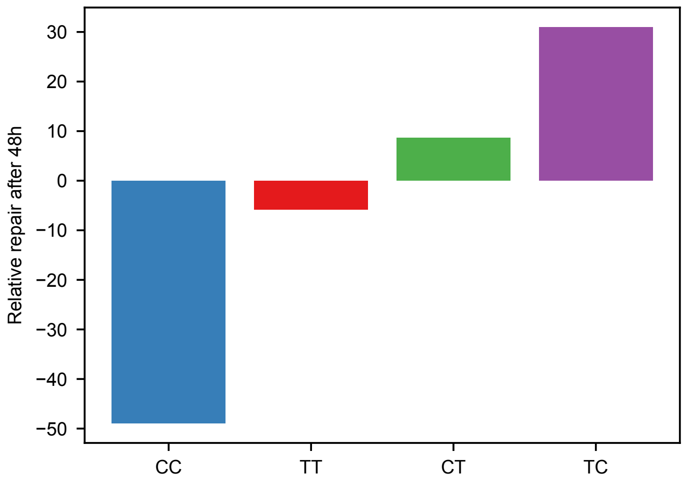

Figure S12. Relative CPDs repair 48h after UV exposure per dipyirimidine type across the whole genome.

Figure S13

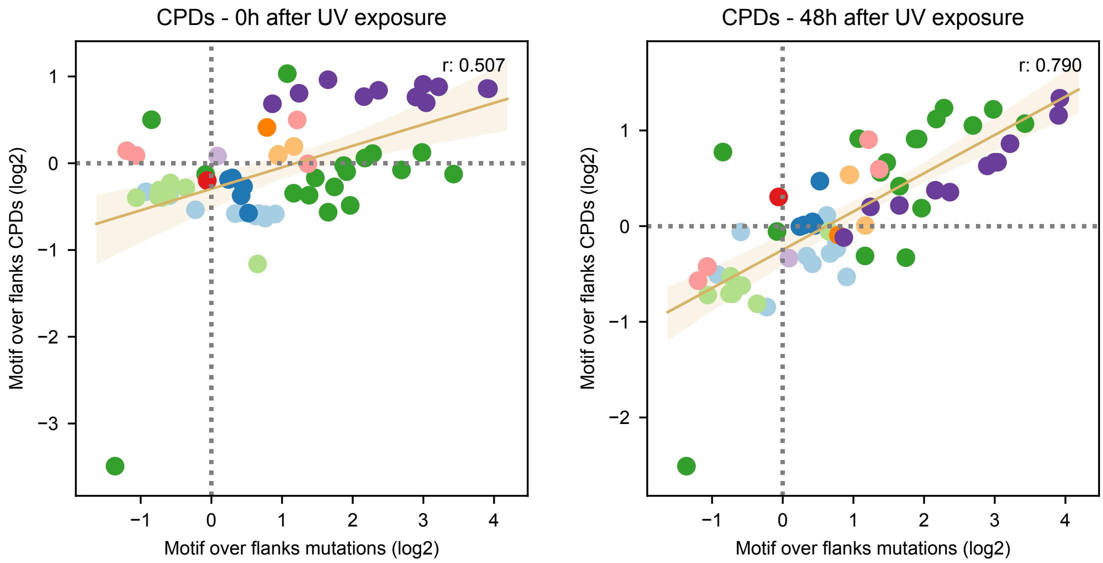

CPDs - 0h after UV exposure
r: 0.507
Motif over flanks CPDs (log2)
Motif over flanks mutations (log2)
CPDs - 48h after UV exposure
r: 0.790
Motif over flanks CPDs (log2)
Motif over flanks mutations (log2)

Figure S13. Relationship between the ratio (log2) of motif-to-flanks CPDs (y-axis) computed immediately after irradiation and the ratio (log2) of motif-to-flanks mutations (x-axis), both 0h (left) and 48h (right) after UV exposure.
